# Gene expression profiles of germ-free and conventional piglets from the same litter

**DOI:** 10.1038/s41598-018-29093-3

**Published:** 2018-07-16

**Authors:** Jing Sun, Hang Zhong, Lei Du, Xiaolei Li, Yuchun Ding, Haoran Cao, Zuohua Liu, Liangpeng Ge

**Affiliations:** 1 Chongqing Academy of Animal Sciences, Chongqing, 402460 China; 2 Key Laboratory of Pig Industry Sciences, Ministry of Agriculture, Chongqing, 402460 China; 3 Chongqing Key Laboratory of Pig Industry Sciences, Chongqing, 402460 China; 4 Institute of Animal Genetics and Breeding, Sichuan Agricultural University, Chengdu, 611130 China

## Abstract

Germ-free (GF) pigs have clear microbiological backgrounds, and are extensively used as large animal models in the biomedical sciences. However, investigations of the transcriptomic differences between GF and cesarean-derived conventional (CV) piglets are limited. To improve our understanding of GF pigs, and to increase the utility of pigs as an alternative non-rodent model, we used RNA sequencing to profile gene expression in five tissues (the oral mucosae, jejunum, colon, liver, and spleen) of four male GF piglets and four male CV piglets from the same litter. We identified 14 genes that were differentially expressed in all five tissues. Seven of these common differentially expressed genes (DEGs) were interferon-inducible genes, and all 14 were consistently downregulated in the GF piglets as compared to the CV piglets. Compared to the other tissues tested, the expression of transcription factors (TFs) in the colon was most affected by the absence of a microbiota. The expression patterns of immune-related genes were downregulated in the GF piglets as compared to the CV piglets, indicating that the intestinal microbiota influenced gene expression in other tissues besides the gut. Gene Ontology (GO) analysis indicated that, in pigs, the intestinal microbiota affected the expression of genes related to immune system function and development.

## Introduction

The anatomical, physiological, and immunological characteristics of the domestic pigs (*Sus scrofa*) closely resemble those of humans^1^ extensively used as large animal models in the modern livestock industry and in the biomedical sciences^2–5^. Indeed, gnotobiotic pig models have been used to evaluate the safety and efficacy human antiviral vaccines^6^, as well as other drugs^7^ and antibiotics^8^. Germ-free (GF) pigs are gnotobiotic pigs that are reared in sterile environments^9^. GF pigs are regarded as clinically relevant models of human diseases, as these pigs manifest similar clinical symptoms to humans and, after human fecal microbiota transplantation, are susceptible to similar intestinal pathogens^10^.

Microbiota play a critical role in the development of the immune system in GF animal models^11^. The spleen is critical for immune system function; comparisons of splenic and intestinal gene expression profiles identified immunity-related crosstalk between the intestine and the immune system of mice^12^. There is also evidence that the liver is involved in immunological function, and that, in the liver, hepatic antigen-presenting cells and innate lymphocytes regulate intracellular immunity tolerance, and autoimmunity^13^. In several taxa, the gut microbiota is important for the normal development of gut-associated immune tissues (GALTs) such as Peyer’s patches and mesenteric lymphoid nodes, as well as for the distribution of lymphoid cells^14–16^. However, with the exception of a single study, where Chordhury *et al*.^17^ investigated gene expression in the small intestinal epitheliums of GF and conventional piglets, little is known about the effects of microbiota on tissue-specific gene expression in GF and cesarean-derived conventional (CV) piglets.

To determine the influence of microbiotas on gene expression profiles in different swine tissue types, RNA-Seq analysis^18^ was performed on five tissues (oral mucosae, jejunum, colon, spleen, and liver) of 25-day-old male GF and CV piglets from the same litter. This study increases the data available for GF piglets, and also enhances our understanding of this important non-rodent model species.

## Results

### Sequencing data summary

We obtained ~70.90 Gb of raw 150-bp paired-end reads: 35.45 Gb from the four GF piglets and 35.45 Gb from the four CV piglets. Using TopHat2 aligner, we successfully mapped an average of 90.35% of the per-sample clean reads back to the *S.scrofa* reference genome^19^ (Sscrofa v10.2; Table 1).

**Table 1:** Quality and characteristics of sequences from cesarean-derived conventional (CV; n = 4) and germ-free (GF; n = 4) piglets from the same litter.

Piglets	Tissue	Raw reads	Clean reads	Clean bases	Clean reads (%)	Error rate (%)	Q20 (%)	Q30 (%)	GC content (%)
GF	Jejunum	48288268	43596668	6.54 G	90.28%	0.02	95.69	90.23	51.30
	Colon	62040000	56094262	8.41 G	90.42%	0.02	95.80	90.44	50.94
	Spleen	46332068	41867278	6.28 G	90.36%	0.02	95.67	90.18	51.68
	Liver	52371044	47750376	7.16 G	91.18%	0.02	95.73	90.13	50.32
	Oral mucosa	52206896	47073820	7.06 G	90.17%	0.02	95.24	89.32	52.38
CV	Jejunum	50648344	43996026	6.6 G	86.87%	0.02	94.64	88.27	51.41
	Colon	49030906	44001630	6.6 G	89.74%	0.02	95.50	89.80	51.22
	Spleen	51290870	47306252	7.1 G	92.23%	0.02	95.59	89.91	52.02
	Liver	56142822	49496190	7.42 G	88.16%	0.02	95.16	89.16	51.40
	Oral mucosa	54799658	51563930	7.73 G	94.10%	0.02	95.87	90.51	52.16

Q20 and Q30 represent the proportion of bases with Phred quality scores >20 and >30, respectively.

### Common differentially expressed genes (DEGs) and transcription factors (TFs) in GF and CV piglets

**Figure 1:**
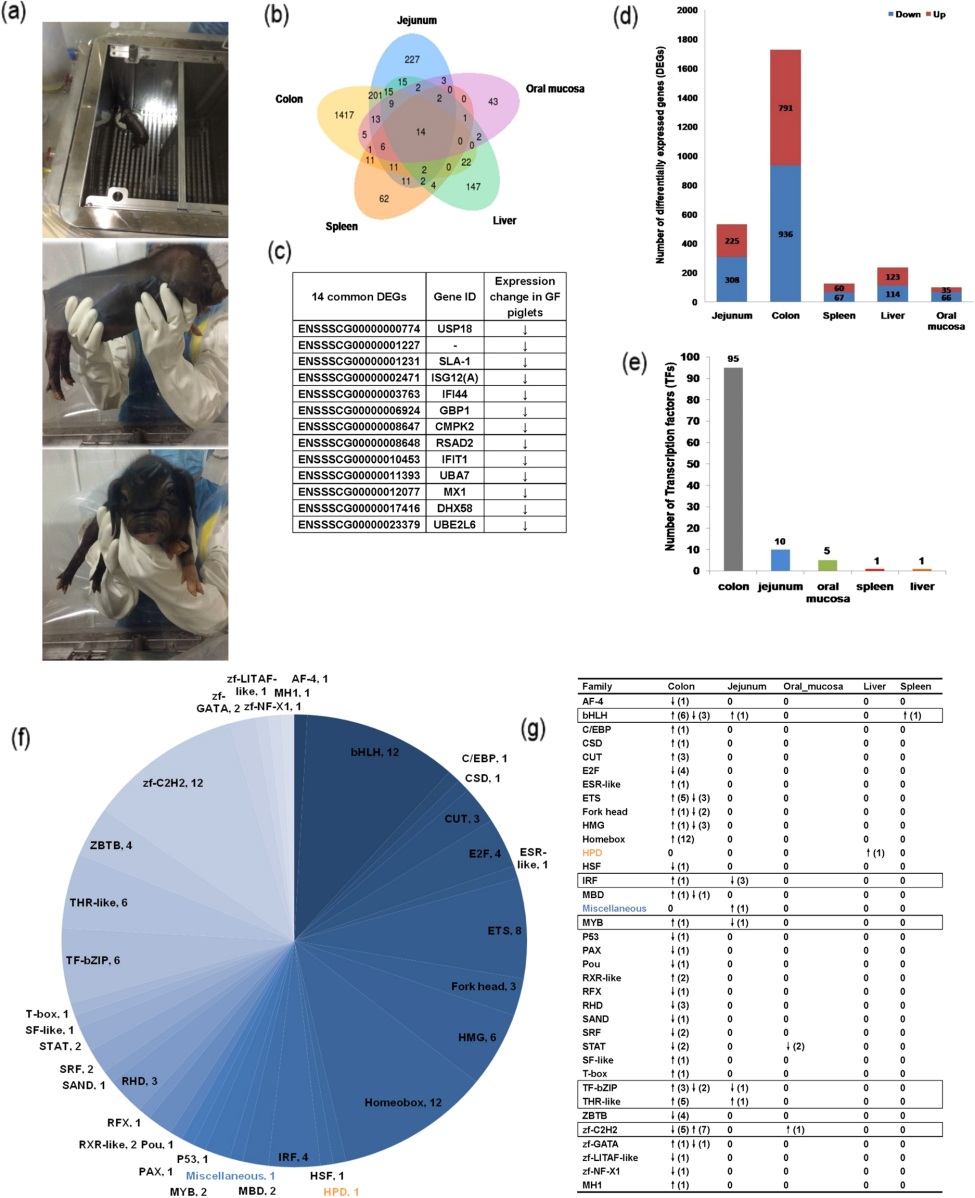
Differential expression of genes and transcription factors (TFs). (**a**) Germ-free piglets in sterile isolators. Distribution of (**b**) differentially expressed genes (DEGs) and (**c**) common DEGs in colons, jejuna, spleens, livers, and oral mucosae of the eight piglets. (**d**) Distribution of up- and downregulated DEGs across all tissues. The (**e**) number and (**f**) distribution of TFs, transcription co-factors, and chromatin remodeling factors (CRFs). (**g**) The distribution of TFs within each tissue. Orange indicates TFs only expressed in the liver, blue indicates TFs only expressed in the jejunum. Black box indicates TFs expressed in two or more tested tissues.

We identified 2,730 DEGs (*P* < 0.001) across all five tissues in both the GF and CV piglets. About 69% of the DEGs were tissue-specific (colon: 1,417; jejunum: 227; liver: 147; spleen: 62; and oral mucosae: 43; Fig. 1b). We identified 14 DEGs expressed in all five tissues from both GF and CV piglets; all 14 of these DEGs were downregulated in the GF piglets as compared to the CV piglets (Fig. 1c).

TFs are important regulators of gene expression in all organisms^20^. We used the refined prediction pipeline in AnimalTFBD^20^ (version 2.0; http://bioinfo.life.hust.edu.cn/AnimalTFDB/) to identify 111 TFs belonging to 36 families in both CV and GF piglets, as well as 46 transcription co-factors and 22 chromatin remodeling factors (CRFs). TF-encoding gene expression patterns differed among the tissues tested (Fig. 1e–g; Supplementary Table S1), with only one TF-encoding gene expressed in the spleen and only one expressed in the liver, but 95 expressed in the colon. More than 84% of all TF-encoding genes were only expressed in the colon, including transcription factors *AF-4*, *E2F*, and *ETS*; domain *Fork head*, domain *Homeobox*, high mobility group box domain (*HMG*), DNA-binding domain (*MBD*), paired domain *PAX*, the cold shock domain (*CSD*) and the homeobox domain (*CUT*); p53 tumor suppressor family (*P53*), CCAAT/enhancer-binding protein (*C/EBP*), the nuclear hormone receptor *RXR-like*, Rel homology domain *RHD*, DNA-binding (Sp100, AIRE-1, Nucp41/75, DEAF-1) *SAND* domain, Nuclear hormone receptor *SF-like*, p53-like transcription factor (*T-box*), BTB/POZ domain (*ZBTB*), SANT-Myb domain (*zf-GATA*), LPS-induced tumor necrosis factor alpha factor (*zf-LITAF-like*), and R3H domain (*zf-NF-X1*). DNA-binding RFX-type winged-helix domain (*RFX*), a gene crucial for ciliogenesis^21^, was only expressed in the jejunum. The TF signal transducer and activator of transcription (STAT), which triggers the production of reactive oxygen species (ROS), was only expressed in the oral mucosa. INF-induced cellular apoptosis is initiated via STAT activation and ROS accumulation^22^. Other TF-encoding genes, including the basic helix-loop-helix (*bHLH*) transcription factor, the basic leucine zipper (*bZIP*) of TF family (*TF-bZIP*), nuclear hormone receptor *THR-like* and Zinc finger (*zf*)*-C2H2*, interferon regulatory factor DNA-binding domain (*IRF*), and Myb-like domain (*MYB*), were expressed in both the colon and jejunum. The bHLH protein is important for the regulation of diverse biological processes, including growth, development, and the stress response^23^. This protein was expressed in the colon, jejunum, and spleen. In addition, we classified 20 novel genes as TF-related (e-value < 1e-5), including 3 genes encoding TFs, 10 genes encoding transcription co-factors, and 7 genes encoding chromatin remodeling factors (CRFs) (Supplementary Table S1).

### Top 30 DEGs from each tissue

We defined DEGs as those where |logFC| was >1 and FDR was <0.005. In the GF piglets as compared to the CV piglets, 10 genes were downregulated and 20 genes were upregulated in the colon; 19 genes were downregulated and 11 were upregulation in the jejunum; 22 genes were downregulated and 8 were upregulated in the spleen; 12 genes were downregulated and 18 were upregulated in the liver; and 18 genes were downregulated and 12 genes were upregulated in the oral mucosa (Supplementary Table S2).

We carefully considered the top 30 genes that were most differentially expressed in GF piglets, as well as the 14 common genes differentially expressed in GF piglets as compared to CV piglets (Fig. 1c; Supplementary Table S2). Of the14 common DEGs identified in all tissues, seven (*ISGA12(A)*, *IFI44*, *GBP1*, *CMPK2*, *IFIT1* and *MX1*) were IFN-inducible genes, two genes (*USP18* and *SLA-1*) were related to the T cell receptor signaling pathway. And all 14 were consistently downregulated in the GF piglets as compared to the CV piglets. As is known, interferon (IFN) is important for antiviral infections through IFN-stimulated genes (ISGs)^24^. *ISG12(A)*, a member of the *ISG12* family, is transcriptionally dysregulated in some disorders^25,26^. *ISG12(A)* was significantly downregulated in the GF piglets as compared to the CV piglets (Fig. 1c; Supplementary Table S2). *SLA-1* (Major histocompatibility complex class I antigen 1 isoform X2) was also downregulated in the jejuna, livers, spleens, and oral mucosae of GF piglets as compared to those of CV piglets. Interestingly, two other *ISGs*, *UBE2L6* (also known as *UBCH8*) and *IFIT1* were downregulated in the oral mucosae of GF piglets as compared to CV piglets (Fig. 1c; Supplementary Table S2).

### Microbiota-induced functional alterations in different tissue types

**Figure 2:**
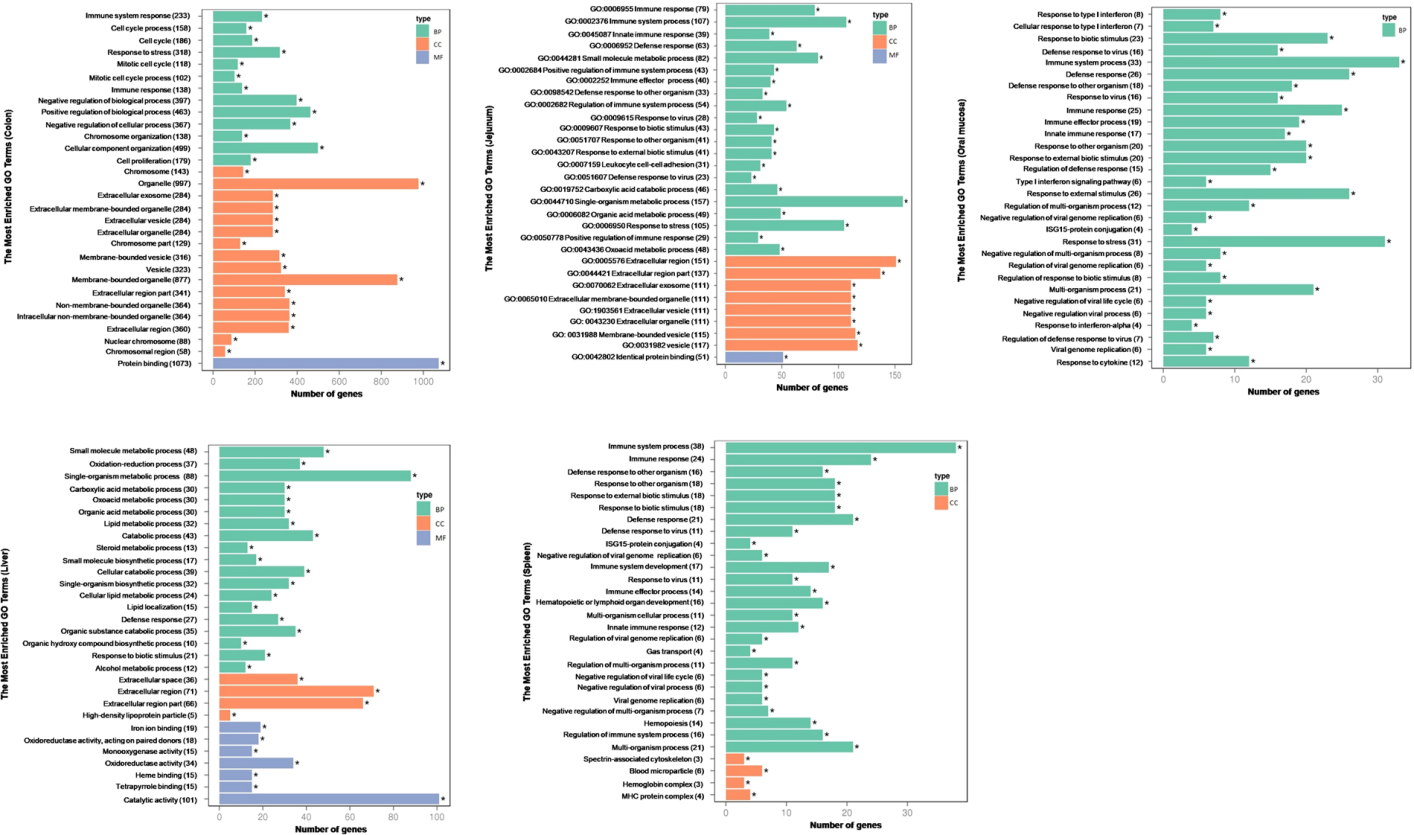
The most enriched Gene Ontology (GO) terms in the colon, jejunum, oral mucosa, liver and spleen. BP: biological process, CC: cellular component, MF: molecular function.

We investigated the biological pathways dysregulated in different tissues based on the Gene Ontology (GO) of the identified DEGs. Approximately 15% of all identified DEGs, except those uniquely expressed in the liver, were functionally associated with the immune system (Fig. 2; Supplementary Table S3). This indicated that the effects regulated by the microbiota extended beyond the gut into tissues such as the spleen, liver, and mucosae. The DEGs identified in the jejunum, colon, spleen, and oral mucosae were involved in defense responses to stress, viruses, and external biotic stimuli, consistent with previous studies showing that microbiotas influence functional aspects of intestinal, mucosal, and systemic immunity^27,28^.

We next calculated the percentage of microbiota-regulated DEGs in each tissue type. DEGs related to the regulation of the immune response were downregulated in the jejuna of GF piglets as compared to CV piglets, whereas those involved in single organism metabolic processes, small molecules, organic acids, and oxoacids were upregulated in the jejuna of GF piglets as compared to CV piglets.

DEGs identified in the oral mucosae of CV piglets were enriched in the type I IFN signaling pathway (Table S4). In contrast, DEGs from the oral mucosae of GF piglets were enriched in ISG15-protein conjugation (Table S4). This functional change suggested that gene expression in the oral mucosae of the piglets was affected by the presence or absence of a microbiota. Type IIFNs, including IFN-α and IFN-β, are products of the innate immune system. In humans, Type I IFNs modulate immune system function^29^ and stimulate natural killer (NK) cells^30^. DEGs associated with several additional immunity related processes (e.g. regulation of viral genome replication and viral defense response) and proteins (e.g. IFN-α and cytokines) were downregulated in the oral mucosae of GF piglets, suggesting that these functions were also affected by the absence of the microbiota (Supplementary Table S3).

### Morphological examination and real-time quantitative PCR (QPCR) confirmation

**Figure 3:**
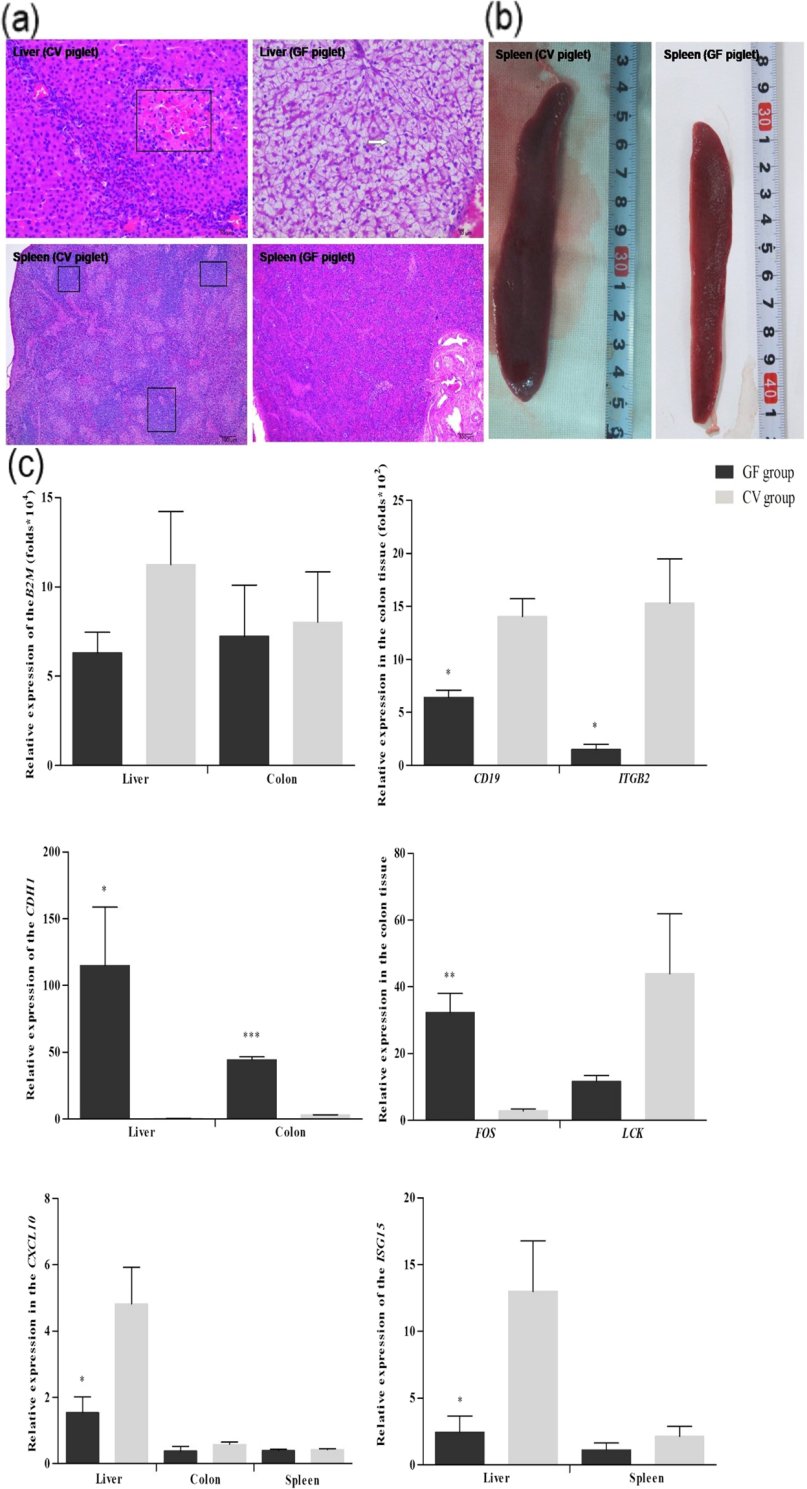
Morphological changes caused by the absence of a microbiota. (**a**) The hematoxylin-eosin (HE) staining results. (**b**) Spleens of germ-free (GF) and cesarean-derived conventional (CV) piglets. (**c**) Validation of DEGs with quantitative real-time PCR (QPCR). In the QPCRs, relative gene expression levels were calculated using the ΔΔCt method and normalized against the reference gene ß-actin. The Y-axis represents fold change. We used unpaired Student’s *t* tests to evaluate the statistical significance of differences between the CV and GF piglets. *P < 0.05; **P < 0.01; ***P < 0.001. All data are presented as means ± standard error (SE).

To investigate the influence of the gut microbiota on piglet morphological development, we compared liver and spleen morphologies between the GF and CV piglets using hematoxylin-eosin (HE) staining (Fig. 3a). Compared with the CV piglets, the GF piglets had smaller spleens (Fig. 3b), and no lymphocytic aggregation was detected in the tissue sections. Hepatocyte morphology was distinctly different between the CV and GF piglets. There was extensive edema in the livers of the GF piglets, while those of the CV piglets had ambiguous cellular outlines coupled with amyloid depositions. We used QPCR to confirm the microbial modulation of the expression of eight exemplar genes in different functional categories (Fig. 3c).

## Discussion

Our results indicated that ~70% of the piglets’ transcriptomes were microbially regulated, primarily including genes that were associated with immunity, response to external controls, and metabolism. The number of DEGs and differentially expressed TFs varied among the tissues tested. The most distinct microbiota-induced alterations in gene expression were observed in the intestinal tissues (colon and jejunum), followed by the immune organs (liver and spleen), and the oral mucosae.

IFNs are a powerful and universal intracellular antiviral defense response system^31^. Of the14 common DEGs identified in all tissues, seven were IFN-inducible genes and two genes including *USP18* and *SLA-1* were related to the T cell receptor signaling pathway. These nine genes were downregulated in the GF piglets as compared to the CV piglets, indicating that commensal bacteria strongly influence the IFN system. USP18, an ISG15 isopeptidase, is a negative regulator of the IFN signaling pathway, which cleaves ISG15-modified proteins^32^. USP18 also increases mice resistance to the vaccinia virus and the influenza B virus *in vivo*^33^.

ISG12 is a critical modulator of the innate immune responses^25^. IGS12 regulates anti-inflammatory nuclear receptors such as NR4A1, which decreases systemic IL6 levels in ISG12-deficient animals^25^. SLA is a component of the major histocompatibility (MHC). As the MHC is vital for maintaining immunological pathogenic resistance^34^, SLA may be involved in disease susceptibility^35^. Because innate immune capacity is reflected by *SLA-1* expression^36^, the significantly upregulated expression of *SLA-1* in CV piglets as compared to GF piglets suggested that gut microbiota improved the function of the piglet immune system.

Guanylate-binding proteins (GBPs) are large IFN-inducible GTPases that are important to the antimicrobial effects of mesenchymal stromal cells (MSCs) on intracellular pathogens and parasites such as *Toxoplasma gondii*^37,38^. Type I IFN is key to innate immunity, stimulating the IFN response pathway to produce antiviral ISG proteins^39^. The interferon-induced proteins with tetratricopeptide repeats (IFITs) are an important subclass of ISG proteins. *IFI44*, a member of the type I IFN-inducible gene family, is potentially related to the inflammations that are associated with gentamicin-induced nephrotoxicity^40^. Various ISG proteins are activated by the IFN response pathway and function as antivirals. *UBE2L6*, an effector of the innate antiviral response^41^, was downregulated in the oral mucosae of the GF piglets as compared to the CV piglets. Drug sensitivity significantly increases after the downregulation of *ISG15* and *UBE2L6*^42^, possibly indicating the potential of these genes as targets for cancer research. Because IFNs have antitumor, antiviral, and anti-proliferative functions, it is clear that the gut microbiota affects the expression of immunity-related genes in piglets.

The piglet microbiotas had a significantly greater impact on TF expression in the gut than in any other the other tissues. TFs regulate and initiate gene expression, and are thus involved in most biological processes^43^. Here, TF-gene expression levels in the colon were strongly affected by the piglet microbiotas. The jejuna, oral mucosae, spleens, and livers were less affected. Specific TFs may thus be potentially useful as pharmaceutical drug targets^43^. In GF piglets, several tumor suppressor TFs, including the *TP53* gene (tumor protein p53) and interferon regulatory factor-1 (*IRF1*), were downregulated in guts of GF piglets as compared to CV piglets, suggesting that microbiotas are essential for the restoration of tumor suppressor activity by these TFs.

STATs are vital for immune system homeostasis. STAT-1 and STAT-3 are particularly important for pathogenic defense^44,45^. Previous studies have shown that *STAT1* knockout mice are susceptible to viral infections^44,46^, and that weaning suppresses the phosphorylation of STAT1 and STAT6 in the jejuna of pigs^45^. Here, STAT1 and STAT2 were suppressed in the colons and oral mucosae of the GF piglets as compared to the CV piglets, suggesting that microbiotal absence was an environmental stressor for the piglets, causing intestinal and mucosal dysfunction along with abnormal immune responses.

Several functional categories related to immunity were enriched in the DEGs, indicating that the absence of a microbiota affected other organs and tissues in addition to the intestine. The DEGs identified in the piglet guts, spleens, and oral mucosae were enriched in immune responses to viruses, stress, and external biotic stimuli, indicating that the microbiota stimulated the expression of relevant genes in these tissues. ISG15, an IFN-induced ubiquitin-like protein that is important in viral infections^47^, was downregulated in all tested tissues of the GF piglets as compared to CV piglets, indicating that the GF piglets had lower antiviral function than the CV piglets. The mucosa-associated lymphoid tissues (MALTs) are the secondary lymphoid organs of the alimentary tract, and act as an immune surveillance system^48^. The oral immune system is a MALT^49^ that defends against infections involving the oral mucosae, salivary glands, and saliva^50^. Here, DEGs associated with the regulation of viral genome replication and with the viral defense response, and as well as with the expression of IFN-alpha and cytokines, were downregulated in GF pigs as compared to CV pigs.

The spleen, a vital lymphoid tissue that removes bacteria from the blood^51^, is underdeveloped in the absence of a microbiota^16,52^. In the spleens of CV piglets, DEGs were enriched in several immune system processes, including system regulation and the response to viruses and other external biotic stimuli. We did not identify similar DEGs in the GF piglets. Indeed, in the spleens of both GF and CV piglets, we identified DEGs related to immunity, including the development of the immune system and of the hematopoietic and lymphoid organs. This suggested that the importance of a microbiota for normal immune system function. The DEGs identified in the livers of CV piglets were involved in single-organism metabolic processes, small molecules, lipids, steroids, alcohols, and the catabolic processes of cellular and organic substances (Table S3), suggesting that the microbiota was essential for proper liver function.

## Conclusions

The microbiota strongly influenced the expression of genes related to the immune system. This study identified 14 genes that were differentially expressed in all five tissues (oral mucosae, jejunum, colon, spleen, and liver). Seven of these common DEGs were IFN-inducible genes, and all 14 were consistently downregulated in the GF piglets as compared to the CV piglets. The highest number of TF genes (whose expressions were) affected by the presence of bacteria was identified in the colon. GO functional analysis indicated that the intestinal microbiota impacted the expression of genes related to immune system development and function in pigs.

## Methods

### Preparation of experimental animals

All animal experiments were conducted pursuant to the Regulations for the Administration of Affairs Concerning Experimental Animals (Ministry of Science and Technology, Beijing, China; revised June 2004). All guidelines related to the care of laboratory animals were followed. The institutional ethics committee of the Chongqing Academy of Animal Science (Chongqing, China) reviewed the relevant ethical issues and approved this study (permit number xky-20150113). All experiments were conducted at the Experimental Swine Engineering Center of the Chongqing Academy of Animal Sciences (CMA No. 162221340234; Rongchang, Chongqing, China).

Eight newborn GF piglets were obtained via hysterectomy from a multiparous Taihu sow (a locally common Chinese pig breed). Four of the piglets (the “GF” group) were kept in a sterile isolator (Class Biologically Clean Ltd., Madison, Wisconsin). The other four piglets (the “CV” group) were fostered by a lactating sow in a conventional agricultural environment. The GF piglets were reared in sterile isolators under GF conditions and were handfed Co60 γ-irradiated sterile 4.8%-fat cow milk powder diluted with sterile water (Anyou Group, Jiangsu, China). Once per week, the GF environments were checked for aerobic and anaerobic bacterial contamination. At 25 days of age, all GF and CV piglets were euthanized under isoflurane anesthesia. The livers, spleens, small intestines, colons, and oral mucosae of all eight piglets were collected and preserved in liquid nitrogen.

### Library preparation for RNA-Seq

Total RNA was extracted using TRIzol reagent (Invitrogen, Carlsbad, CA, USA), following the manufacturer’s instructions. RNA degradation and contamination were monitored using 1% agarose gels. RNA purity was checked using a Nano Photometer spectrophotometer (Implen, Los Angeles, CA, USA). RNA concentration was measured using a Qubit RNA Assay Kit in a Qubit2.0 Fluorometer (Life Technologies, Carlsbad, CA, USA). RNA integrity was assessed using an RNA Nano 6000 Assay Kit on a Bioanalyzer 2100 (Agilent Technologies, Santa Clara, CA, USA). We used ~3 μg of total RNA per sample as input material for the construction of sequencing libraries. Libraries were generated using the NEB Next Ultra RNA Library Prep Kit for Illumina (NEB, USA), following the manufacturer’s instructions. Index codes were added to connect sequences to samples. The quality of all sequences was assessed with an Agilent Bioanalyzer 2100.

We pooled the samples from each tissue by group and prepared a total of 10 sequencing libraries. All libraries were sequenced on an Illumina HiSeq. 4000 platform, generating 150-bp paired-end reads.

### Data processing and DEG identification

High-quality clean reads were obtained from raw reads by trimming adapter sequences, removing invalid reads containing poly-N, and eliminating low-quality reads. The clean reads were mapped to the *Sus scrofa* genome (*Sus scrofa* 10.2, FA; ftp://ftp.ensembl.org/pub/release-81/fasta/sus_scrofa/dna/) using TopHat (v2.0.12) with default parameters (mismatch = 2). Novel transcripts were predicted by comparing reconstructed transcripts with known transcripts using Cufflinks (v2.1.1)^53^. Gene expression was measured with HTSeq^54^ (v0.6.1), and normalized using the expected number of fragments per kilobase of transcript sequence per millions base pairs sequenced (FPKM) method^55^. DEGs were identified using the DEGSeq package^56^ (v1.20.0), which was designed to be used without biological replicates. P values were adjusted using the Benjamini & Hochberg method of DEF screening: we considered FDR ≤ 0.01 and an absolute value of log_2_(fold change) ≥ 1 to indicate a significant difference in gene expression.

### GO enrichment analysis of DEGs

We used the GOseq R package^57^, which corrects for gene length bias, to analyze the GO enrichment of the identified DEGs. GO terms with Q-value < 0.05 were considered significantly enriched.

### Validation of DEGs and morphological comparisons

Eight DEGs were randomly selected for further confirmation using real-time quantitative PCR (QPCR) (the primers used are listed in Supplementary Table S4). We examined all piglet livers and spleens after HE staining to identify morphological differences between the GF and the CV piglets. Histological images were analyzed.

## Electronic supplementary material

Table S1

Table S2

Table S3

Table S4

## References

[1] Wang M, Donovan SM (2015). Human microbiota-associated swine: current progress and future opportunities. ILAR journal.

[2] Powell EJ, Cunnick JE, Tuggle CK (2017). SCID pigs: An emerging large animal NK model. Journal of rare diseases research & treatment.

[3] Suzuki, T. *et al*. Application of actuator-driven pulsed water jet for coronary artery bypass grafting: assessment in a swine model. *Journal of artificial organs: the official journal of the Japanese Society for Artificial Organs*, 10.1007/s10047-017-1008-z (2017). 10.1007/s10047-017-1008-z 29147809

[4] Chardon P, Renard C, Vaiman M (1999). The major histocompatibility complex in swine. Immunological reviews.

[5] Niemann H, Kues WA (2003). Application of transgenesis in livestock for agriculture and biomedicine. Animal reproduction science.

[6] Azevedo MP, Vlasova AN, Saif LJ (2013). Human rotavirus virus-like particle vaccines evaluated in a neonatal gnotobiotic pig model of human rotavirus disease. Expert review of vaccines.

[7] Jung K (2012). The effects of simvastatin or interferon-alpha on infectivity of human norovirus using a gnotobiotic pig model for the study of antivirals. Plos One.

[8] Zhang Q (2009). Gnotobiotic piglet infection model for evaluating the safe use of antibiotics against *Escherichia coli* O157:H7 infection. The Journal of infectious diseases.

[9] Meyer RC, Bohl EH, Kohler EM (1964). Procurement And Maintenance Of Germ-Free Seine for Microbiological Investigations. Applied microbiology.

[10] Zhang Q, Widmer G, Tzipori S (2013). A pig model of the human gastrointestinal tract. Gut microbes.

[11] Tlaskalov (2011). The role of gut microbiota (commensal bacteria) and the mucosal barrier in the pathogenesis of inflammatory and autoimmune diseases and cancer: contribution of germ-free and gnotobiotic animal models of human diseases. Cellular & Molecular Immunology.

[12] Masahiro Y (2012). A microarray analysis of gnotobiotic mice indicating that microbial exposure during the neonatal period plays an essential role in immune system development. Bmc Genomics.

[13] Doherty DG (2016). Immunity, tolerance and autoimmunity in the liver: A comprehensive review. Journal of autoimmunity.

[14] Potockova H, Sinkorova J, Karova K, Sinkora M (2015). The distribution of lymphoid cells in the small intestine of germ-free and conventional piglets. Developmental and comparative immunology.

[15] Rothkotter HJ, Pabst R (1989). Lymphocyte subsets in jejunal and ileal Peyer’s patches of normal and gnotobiotic minipigs. Immunology.

[16] Jing S (2016). Measurement of body weight, blood parameters and main organ coefficients of germ-free piglets. Acta Laboratorium Animalis Scientia Sinica.

[17] Chowdhury SR (2007). Transcriptome profiling of the small intestinal epithelium in germfree versus conventional piglets. BMC genomics.

[18] Wang Z, Gerstein M, Snyder M (2009). RNA-Seq: a revolutionary tool for transcriptomics. Nature reviews. Genetics.

[19] Groenen MA (2012). Analyses of pig genomes provide insight into porcine demography and evolution. Nature.

[20] Zhang HM (2015). AnimalTFDB 2.0: a resource for expression, prediction and functional study of animal transcription factors. Nucleic acids research.

[21] Piasecki BP, Burghoorn J, Swoboda P (2010). Regulatory Factor X (RFX)-mediated transcriptional rewiring of ciliary genes in animals. Proceedings of the National Academy of Sciences of the United States of America.

[22] Wang, Y. *et al*. The STAT-ROS cycle extends IFNinduced cancer cell apoptosis. *International journal of oncology*, 10.3892/ijo.2017.4196 (2017). 10.3892/ijo.2017.4196 29115415

[23] Niu X, Guan Y, Chen S, Li H (2017). Genome-wide analysis of basic helix-loop-helix (bHLH) transcription factors in Brachypodium distachyon. BMC genomics.

[24] Schneider WM, Chevillotte MD, Rice CM (2014). Interferon-stimulated genes: a complex web of host defenses. Annual review of immunology.

[25] Uhrin P, Perkmann T, Binder B, Schabbauer G (2013). ISG12 is a critical modulator of innate immune responses in murine models of sepsis. Immunobiology.

[26] Kim MS, Min KS, Imakawa K (2013). Regulation of Interferon-stimulated Gene (ISG)12, ISG15, and MX1 and MX2 by Conceptus Interferons (IFNTs) in Bovine Uterine Epithelial Cells. Asian-Australasian journal of animal sciences.

[27] Lee YK, Mazmanian SK (2010). Has the microbiota played a critical role in the evolution of the adaptive immune system?. Science.

[28] Sonnenberg GF, Artis D (2012). Innate lymphoid cell interactions with microbiota: implications for intestinal health and disease. Immunity.

[29] Brod SA (1999). Autoimmunity is a type I interferon-deficiency syndrome corrected by ingested type I IFN via the GALT system. Journal of interferon & cytokine research: the official journal of the International Society for Interferon and Cytokine Research.

[30] Nguyen KB (2002). Coordinated and distinct roles for IFN-alpha beta, IL-12, and IL-15 regulation of NK cell responses to viral infection. Journal of immunology.

[31] Haller O, Kochs G, Weber F (2007). Interferon, Mx, and viral countermeasures. Cytokine & growth factor reviews.

[32] Basters, A., Knobeloch, K. P. & Fritz, G. How USP18 deals with ISG15-modified proteins: structural basis for the specificity of the protease. *The FEBS journal*10.1111/febs.14260 (2017). 10.1111/febs.14260 28881486

[33] Ketscher L (2015). Selective inactivation of USP18 isopeptidase activity *in vivo* enhances ISG15 conjugation and viral resistance. Proceedings of the National Academy of Sciences of the United States of America.

[34] Pedersen LE, Jungersen G, Sorensen MR, Ho CS, Vadekaer DF (2014). Swine Leukocyte Antigen (SLA) class I allele typing of Danish swine herds and identification of commonly occurring haplotypes using sequence specific low and high resolution primers. Veterinary immunology and immunopathology.

[35] Rothsehild, M. F., Skow, L. & SJ, L. In *Breeding for disease resistance in farm animals* 73–105 (CABI Publishing, 2000).

[36] Ye L (2012). Investigation of the relationship between SLA-1 and SLA-3 gene expression and susceptibility to Escherichia coli F18 in post-weaning pigs. Comparative immunology, microbiology and infectious diseases.

[37] Johnston AC (2016). Human GBP1 does not localize to pathogen vacuoles but restricts Toxoplasma gondii. Cellular microbiology.

[38] Spekker K (2013). Antimicrobial effects of murine mesenchymal stromal cells directed against Toxoplasma gondii and Neospora caninum: role of immunity-related GTPases (IRGs) and guanylate-binding proteins (GBPs). Medical microbiology and immunology.

[39] Rabbani MA, Ribaudo M, Guo JT, Barik S (2016). Identification of Interferon-Stimulated Gene Proteins That Inhibit Human Parainfluenza Virus Type 3. Journal of virology.

[40] Hu JG (2017). Altered gene expression profile in a rat model of gentamicin-induced ototoxicity and nephrotoxicity, and the potential role of upregulated Ifi44 expression. Molecular medicine reports.

[41] Young DF (2016). Human IFIT1 Inhibits mRNA Translation of Rubulaviruses but Not Other Members of the Paramyxoviridae Family. Journal of virology.

[42] Falvey CM (2017). UBE2L6/UBCH8 and ISG15 attenuate autophagy in esophageal cancer cells. Oncotarget.

[43] Ulasov AV, Rosenkranz AA, Sobolev AS (2017). Transcription factors: Time to deliver. Journal of controlled release: official journal of the Controlled Release Society.

[44] Bradfute SB, Stuthman KS, Shurtleff AC, Bavari S (2011). A STAT-1 knockout mouse model for Machupo virus pathogenesis. Virology journal.

[45] Yi H (2016). Developmental expression of STATs, nuclear factor-kappaB and inflammatory genes in the jejunum of piglets during weaning. International immunopharmacology.

[46] Raymond J, Bradfute S, Bray M (2011). Filovirus infection of STAT-1 knockout mice. The Journal of infectious diseases.

[47] Morales DJ, Lenschow DJ (2013). The antiviral activities of ISG15. Journal of molecular biology.

[48] Sipos F, Muzes G (2011). Isolated lymphoid follicles in colon: switch points between inflammation and colorectal cancer?. World journal of gastroenterology.

[49] Czerkinsky C (1999). Mucosal immunity and tolerance: relevance to vaccine development. Immunological reviews.

[50] Walker DM (2004). Oral mucosal immunology: an overview. Annals of the Academy of Medicine, Singapore.

[51] Altamura M (2001). Splenectomy and sepsis: the role of the spleen in the immune-mediated bacterial clearance. Immunopharmacology and immunotoxicology.

[52] Hegde, S. N., Rolls, B. A., Turvey, A. & E Coates, M. *Influence of gut microflora on the lymphoid tissue of the chicken (Gallus domesticus) and Japanese quail (Coturnix coturnix Japonica)*. Vol. 72 (1982).

[53] Trapnell C (2012). Differential gene and transcript expression analysis of RNA-seq experiments with TopHat and Cufflinks. Nature protocols.

[54] Anders S, Pyl PT, Huber W (2015). HTSeq–a Python framework to work with high-throughput sequencing data. Bioinformatics.

[55] Trapnell C (2010). Transcript assembly and quantification by RNA-Seq reveals unannotated transcripts and isoform switching during cell differentiation. Nature biotechnology.

[56] Wang L, Feng Z, Wang X, Wang X, Zhang X (2010). DEGseq: an R package for identifying differentially expressed genes from RNA-seq data. Bioinformatics.

[57] Young MD, Wakefield MJ, Smyth GK, Oshlack A (2010). Gene ontology analysis for RNA-seq: accounting for selection bias. Genome biology.

