# Supplementary material for: Gene expression profiles of germ-free and conventional piglets from the same litter
Source: Sci Rep. 2018 Jul 16;8:10745. doi: 10.1038/s41598-018-29093-3 (PMC6048018; doi:10.1038/s41598-018-29093-3)
Supplement: Supplementary file 1 — Table S1 [file 41598_2018_29093_MOESM1_ESM.pdf]

Gene expression profiles of germ-free and conventional piglets from the same litter

Jing Sun[1,2,3,*], Hang Zhong[1,*], Lei Du[1], XiaoLei Li[1,4], Yuchun Ding[1,2,3], Haoran Cao[1,2,3], Zuohua Liu[1,2,3,+], and Liangpeng Ge[1,2,3,+]

Table S1. List of 199 genes (including 20 novel genes) and their transcription factors.

**Gene ID**	**Family**	**Gene name**	**Regulated in GF piglets**	**tissue**	**Description**
Novel01919	MYB	*MYB*	↓	Colon	ENSSSCG00000004167 symbol:MYB family:MYB species:Sus scrofa
Novel02271	ETS	*SPI1*	↓	Colon	ENSSSCG00000013237 symbol:SPI1 family:ETS species:Sus scrofa
Novel02379	TF_bZIP	*MAFB*	↑	Colon	ENSSSCG00000030898 symbol:MAFB family:TF_bZIP species:Sus scrofa
Novel01097	Transcription Cofactors	*RHOA*	↑	Colon	ENSSSCG00000022080 symbol:RHOA family:Transcription Cofactors species:Sus scrofa
Novel01281	Transcription Cofactors	*ABL1*	↓	Colon	ENSSSCG00000005706 symbol:ABL1 family:Transcription Cofactors species:Sus scrofa
Novel01362	Transcription Cofactors	-	↓	Colon	ENSSSCG00000001932 family:Transcription Cofactors species:Sus scrofa
Novel01770	Transcription Cofactors	*TRIM27*	↑	Colon	ENSSSCG00000001219 symbol:TRIM27 family:Transcription Cofactors species:Sus scrofa
Novel01879	Transcription Cofactors	*TRIM27*	↑	Colon	ENSSSCG00000001219 symbol:TRIM27 family:Transcription Cofactors species:Sus scrofa
Novel02108	Transcription Cofactors	*RAN*	↑	Colon	ENSSSCG00000009746 symbol:RAN family:Transcription Cofactors species:Sus scrofa

Novel02542	Transcription Cofactors	*TRIM27*	↓	Colon	ENSSSCG00000001219 symbol:TRIM27 family:Transcription Cofactors species:Sus scrofa
Novel03111	Transcription Cofactors	*IL16*	↑	Colon	ENSSSCG00000001787 symbol:IL16 family:Transcription Cofactors species:Sus scrofa
Novel03261	Transcription Cofactors	*LOXL4*	↑	Colon	ENSSSCG00000010532 symbol:LOXL4 family:Transcription Cofactors species:Sus scrofa
Novel03459	Transcription Cofactors	*ABL1*	↑	Colon	ENSSSCG00000005706 symbol:ABL1 family:Transcription Cofactors species:Sus scrofa
Novel00120	Chromatin Remodeling Factors	*MTF2*	↓	Colon	ENSSSCG00000006898 symbol:MTF2 family:Chromatin Remodeling Factors species:Sus scrofa
Novel00523	Chromatin Remodeling Factors	*CHD4*	↓	Colon	ENSSSCG00000000697 symbol:CHD4 family:Chromatin Remodeling Factors species:Sus scrofa
Novel01281	Chromatin Remodeling Factors	*JAK2*	↓	Colon	ENSSSCG00000005215 symbol:JAK2 family:Chromatin Remodeling Factors species:Sus scrofa
Novel01352	Chromatin Remodeling Factors	*EHMT2*	↓	Colon	ENSSSCG00000001420 symbol:EHMT2 family:Chromatin Remodeling Factors species:Sus scrofa
Novel01484	Chromatin Remodeling Factors	*SMARCA2*	↓	Colon	ENSSSCG00000005232 symbol:SMARCA2 family:Chromatin Remodeling Factors

					species:Sus scrofa
Novel02117	Chromatin Remodeling Factors	*HDAC4*	↓	Colon	ENSSSCG00000024777 symbol:HDAC4 family:Chromatin Remodeling Factors species:Sus scrofa
Novel03155	Chromatin Remodeling Factors	*HDAC4*	↓	Colon	ENSSSCG00000024777 symbol:HDAC4 family:Chromatin Remodeling Factors species:Sus scrofa
ENSSSCG00000012730	AF-4	*AFF2*	↓	Colon	Transcription factor AF4/FMR2
ENSSSCG00000004174	bHLH	*TCF21*	↑	Spleen	Myc-type, basic helix-loop-helix (bHLH) domain
ENSSSCG00000007227	bHLH	*ID1*	↑	Colon	Myc-type, basic helix-loop-helix (bHLH) domain
ENSSSCG00000007710	bHLH	*MLXIPL*	↑	Colon	Myc-type, basic helix-loop-helix (bHLH) domain
ENSSSCG00000008645	bHLH	*ID2*	↑	Colon	Myc-type, basic helix-loop-helix (bHLH) domain
ENSSSCG00000013437	bHLH	*TCF3*	↓	Colon	Myc-type, basic helix-loop-helix (bHLH) domain
ENSSSCG00000013743	bHLH	-	↓	Colon	Myc-type, basic helix-loop-helix (bHLH) domain
ENSSSCG00000014041	bHLH	*MXD3*	↓	Colon	Myc-type, basic helix-loop-helix (bHLH) domain
ENSSSCG00000024312	bHLH	*ID4*	↑	Colon	Myc-type, basic helix-loop-helix (bHLH) domain||Tryptophan synthase beta subunit-like PLP-dependent enzyme
ENSSSCG00000022128	bHLH	*MXI1*	↑	Colon	Myc-type, basic helix-loop-helix (bHLH) domain
ENSSSCG00000026362	bHLH	*HES1*	↑	Colon	Myc-type, basic helix-loop-helix (bHLH)

					domain‖Orange domain
ENSSSCG00000023124	bHLH	*SREBF1*	↑	Jejunum	Myc-type, basic helix-loop-helix (bHLH) domain
ENSSSCG00000002866	C/EBP	*CEBPA*	↑	Colon	CCAAT/enhancer-binding protein, chordates‖Basic-leucine zipper domain
ENSSSCG00000000633	CSD	*YBX3*	↑	Colon	Cold-shock protein, DNA-binding‖Nucleic acid-binding, OB-fold‖Cold shock protein
ENSSSCG00000011201	CUT	*SATB1*	↑	Colon	Homeodomain-like‖CUT domain‖Homeobox domain‖Lambda repressor-like, DNA-binding domain
ENSSSCG00000021657	CUT	-	↑	Colon	CUT domain‖Homeobox domain‖Lambda repressor-like, DNA-binding domain‖Homeodomain-like
ENSSSCG00000030990	CUT	*SATB2*	↑	Colon	CUT domain‖-‖Lambda repressor-like, DNA-binding domain
ENSSSCG00000002787	E2F	*E2F4*	↓	Colon	E2F/DP family, winged-helix DNA-binding domain
ENSSSCG00000007268	E2F	*E2F1*	↓	Colon	E2F/DP family, winged-helix DNA-binding domain
ENSSSCG00000009560	E2F	-	↓	Colon	Transcription factor DP‖E2F/DP family, winged-helix DNA-binding domain‖Transcription factor DP, C-terminal
ENSSSCG00000013352	E2F	*E2F8*	↓	Colon	E2F/DP family, winged-helix DNA-binding domain
ENSSSCG00000024987	ESR-like	*NR3C2*	↑	Colon	Nuclear hormone receptor‖Nuclear hormone

					receptor, ligand-binding domain‖Zinc finger, nuclear hormone receptor-type
ENSSSCG00000010922	ETS	*ELF3*	↑	Colon	Sterile alpha motif/pointed domain‖Ets domain‖Pointed domain
ENSSSCG00000012068	ETS	*ETS2*	↑	Colon	Pointed domain‖Ets domain‖Sterile alpha motif/pointed domain
ENSSSCG00000013300	ETS	*EHF*	↑	Colon	Sterile alpha motif/pointed domain‖Pointed domain‖Ets domain
ENSSSCG00000015235	ETS	*ETS1*	↓	Colon	Sterile alpha motif/pointed domain‖Ets domain‖Transforming protein C-ets‖Pointed domain
ENSSSCG00000024531	ETS	*SPDEF*	↑	Colon	Ets domain‖Pointed domain‖Sterile alpha motif/pointed domain
ENSSSCG00000025182	ETS	*ELK4*	↓	Colon	Ets domain
ENSSSCG00000024591	ETS	*ETV1*	↑	Colon	Ets domain‖PEA3-type ETS-domain transcription factor, N-terminal
ENSSSCG00000013237	ETS	*SPI1*	↓	Colon	Ets domain
ENSSSCG00000000739	Fork head	*FOXM1*	↓	Colon	Fork head domain
ENSSSCG00000001940	Fork head	*FOXA1*	↑	Colon	Forkhead box protein, C-terminal
ENSSSCG00000009370	Fork head	*FOXO1*	↓	Colon	Fork head domain
ENSSSCG00000001081	HMG	*SOX4*	↑	Colon	High mobility group box domain
ENSSSCG00000008682	HMG	*WHSC1*	↓	Colon	Zinc finger, PHD-type
ENSSSCG00000009327	HMG	*HMGB1*	↓	Colon	High mobility group box domain
ENSSSCG00000005052	HMG	*WDHD1*	↓	Colon	Translation initiation factor, beta propellor-like domain
ENSSSCG0000	HMG	-	↓	Colon	High mobility group box

0022459					domain
ENSSSCG00000000153	Homeobox	*ISX*	↑	Colon	Homeobox domain
ENSSSCG00000005604	Homeobox	*PBX3*	↑	Colon	Homeobox domain
ENSSSCG00000009317	Homeobox	*CDX2*	↑	Colon	Homeodomain-like
ENSSSCG00000009919	Homeobox	*HNF1A*	↑	Colon	Hepatocyte nuclear factor 1, beta isoform, C-terminal
ENSSSCG00000016698	Homeobox	*HOXA11*	↑	Colon	Domain of unknown function DUF3528, homeobox protein, eukaryotic
ENSSSCG00000016700	Homeobox	-	↑	Colon	Hox9, N-terminal activation domain
ENSSSCG00000016702	Homeobox	*HOXA6*	↑	Colon	Homeobox protein, antennapedia type
ENSSSCG00000016703	Homeobox	*HOXA5*	↑	Colon	Homeodomain, metazoa
ENSSSCG00000017536	Homeobox	*HOXB9*	↑	Colon	Hox9, N-terminal activation domain
ENSSSCG00000017538	Homeobox	*HOXB7*	↑	Colon	Homeobox protein, antennapedia type
ENSSSCG00000017539	Homeobox	*HOXB6*	↑	Colon	Homeobox protein, antennapedia type
ENSSSCG00000023417	Homeobox	-	↑	Colon	Homeodomain-like
ENSSSCG00000015584	HPD	*PROX1*	↑	Liver	Homeo-prospero domain
ENSSSCG00000002792	HSF	*HSF4*	↓	Colon	-||Heat shock factor (HSF)-type, DNA-binding
ENSSSCG00000002002	IRF	*IRF9*	↓	Jejunum	Interferon regulatory factor-3
ENSSSCG00000014277	IRF	*IRF1*	↓	Jejunum	Interferon regulatory factor-1/2
ENSSSCG00000015612	IRF	*IRF6*	↑	Colon	Interferon regulatory factor-3
ENSSSCG00000022547	IRF	-	↓	Jejunum	Interferon regulatory factor-3
ENSSSCG00000004528	MBD	*MBD2*	↓	Colon	DNA-binding domain
ENSSSCG00000015882	MBD	*BAZ2B*	↑	Colon	DDT domain, subgroup

ENSSSCG00000004952	MH1	*SMAD3*	↑	Colon	SMAD/FHA domain
ENSSSCG00000003565	Miscellaneous	*NROB2*	↑	Jejunum	Nuclear hormone receptor
ENSSSCG00000007366	MYB	*MYBL2*	↓	Jejunum	SANT/Myb domain
ENSSSCG00000016920	MYB	*MIER3*	↑	Colon	ELM2 domain||Homeodomain-like||SANT/Myb domain
ENSSSCG00000017950	P53	*TP53*	↓	Colon	p53 tumour suppressor family
ENSSSCG00000005345	PAX	*PAX5*	↓	Colon	Paired domain
ENSSSCG00000028237	Pou	*POU2F3*	↓	Colon	POU-specific domain
ENSSSCG00000002254	RXR-like	*NR2F2*	↑	Colon	Transcription factor COUP
ENSSSCG00000007371	RXR-like	*HNF4A*	↑	Colon	Transcription factor COUP
ENSSSCG00000006625	RFX	*RFX5*	↓	Colon	DNA-binding RFX-type winged-helix domain
ENSSSCG00000004856	RHD	-	↓	Colon	Nuclear factor of activated T cells (NFAT)
ENSSSCG00000008388	RHD	*REL*	↓	Colon	IPT domain
ENSSSCG00000007477	RHD	*NFATC2*	↓	Colon	IPT domain
ENSSSCG00000016262	SAND	*SP140*	↓	Colon	Zinc finger, FYVE/PHD-type
ENSSSCG00000014149	SRF	*MEF2C*	↓	Colon	Transcription factor, MADS-box
ENSSSCG00000030351	SRF	*BORCS8-MEF2B*	↓	Colon	Transcription factor, MADS-box
ENSSSCG00000000396	STAT	*STAT2*	↓	Colon and Oral mucosa	STAT transcription factor, protein interaction
ENSSSCG00000016057	STAT	*STAT1*	↓	Colon and Oral mucosa	STAT transcription factor, all-alpha domain
ENSSSCG00000024975	SF-like	*NR5A2*	↑	Colon	Nuclear hormone receptor, ligand-binding domain
ENSSSCG0000	T-box	*TBX2*	↑	Colon	p53-like transcription

0027060					factor, DNA-binding
ENSSSCG00000002383	TF_bZIP	*FOS*	↑	Colon	Transcription factor, Skn-1-like, DNA-binding domain
ENSSSCG00000004332	TF_bZIP	*BACH2*	↓	Colon	Basic-leucine zipper domain
ENSSSCG00000011106	TF_bZIP	*CREM*	↓	Colon	Coactivator CBP, pKID
ENSSSCG00000013263	TF_bZIP	*CREB3L1*	↑	Colon	Transcription factor, Skn-1-like, DNA-binding domain
ENSSSCG00000013501	TF_bZIP	*CREB3L3*	↑	Colon	Transcription factor, Skn-1-like, DNA-binding domain
ENSSSCG00000023178	TF_bZIP	*BATF2*	↓	JEJ	Basic-leucine zipper domain
ENSSSCG00000000875	THR-like	*NR1H4*	↑	Colon	Nuclear hormone receptor
ENSSSCG00000006353	THR-like	*NR1I3*	↑	Jejunum	Nuclear hormone receptor, ligand-binding domain
ENSSSCG00000011211	THR-like	*THRB*	↑	Colon	Thyroid hormone receptor
ENSSSCG00000011579	THR-like	*PPARG*	↑	Colon	Peroxisome proliferator-activated receptor, gamma
ENSSSCG00000011890	THR-like	*NR1I2*	↑	Colon	Zinc finger, nuclear hormone receptor-type
ENSSSCG00000020864	THR-like	*VDR*	↑	Colon	Nuclear hormone receptor
ENSSSCG00000002908	ZBTB	*ZBTB32*	↓	Colon	BTB/POZ domain
ENSSSCG00000011810	ZBTB	*BCL6*	↓	Colon	BTB/POZ domain
ENSSSCG00000030070	ZBTB	*ZBTB18*	↓	Colon	Zinc finger, C2H2-like
ENSSSCG00000026636	ZBTB	*ZBTB4*	↓	Colon	Zinc finger, C2H2-like
ENSSSCG00000007526	zf-C2H2	*ZNF831*	↓	Colon	Zinc finger, C2H2-like
ENSSSCG00000009462	zf-C2H2	-	↑	Colon	Zinc finger, C2H2-like
ENSSSCG00000010911	zf-C2H2	*ZNF281*	↓	Colon	Zinc finger, C2H2-like
ENSSSCG0000	zf-C2H2	*MECOM*	↑	Colon	Zinc finger, C2H2

0011743					
ENSSSCG00000014336	zf-C2H2	*EGR1*	↑	Oral mucosa	Zinc finger, C2H2-like
ENSSSCG00000016164	zf-C2H2	*IKZF2*	↓	Colon	Zinc finger, C2H2
ENSSSCG00000017494	zf-C2H2	*IKZF3*	↓	Colon	Zinc finger, C2H2
ENSSSCG00000029806	zf-C2H2	-	↓	Colon	Zinc finger, C2H2
ENSSSCG00000022270	zf-C2H2	*KLF3*	↑	Colon	Zinc finger, C2H2-like
ENSSSCG00000027361	zf-C2H2	*OVOL1*	↑	Colon	Zinc finger, C2H2-like
ENSSSCG00000023653	zf-C2H2	*GLIS2*	↑	Colon	Zinc finger, C2H2
ENSSSCG00000023349	zf-C2H2	*ZNF768*	↑	Colon	Zinc finger, C2H2-like
ENSSSCG00000005437	zf-C2H2	*KLF4*	↑	Colon	Zinc finger, C2H2
ENSSSCG00000003702	zf-GATA	*GATA6*	↑	Colon	GATA-type transcription activator, N-terminal
ENSSSCG00000013062	zf-GATA	*MTA2*	↓	Colon	SANT/Myb domain
ENSSSCG00000007941	zf-LITAF-like	*CDIP1*	↓	Colon	LPS-induced tumour necrosis factor alpha factor
ENSSSCG00000010993	zf-NF-X1	-	↓	Colon	R3H domain
ENSSSCG00000000373	Transcription Cofactors	*CDK2*	↓	Colon	Protein kinase domain
ENSSSCG00000000745	Transcription Cofactors	*DDX11*	↓	Colon	DEAD2
ENSSSCG00000001075	Transcription Cofactors	*DEK*	↓	Colon	SAP domain
ENSSSCG00000001095	Transcription Cofactors	*GMNN*	↓	Colon	Geminin/Multicilin
ENSSSCG00000001509	Transcription Cofactors	*DAXX*	↓	Colon	Daxx, N-terminal Rassf1C-interacting domain
ENSSSCG00000001787	Transcription Cofactors	*IL16*	↓	Colon	PDZ domain
ENSSSCG00000001912	Transcription Cofactors	*PML*	↓	Jejunum	Protein of unknown function DUF3583
ENSSSCG0000	Transcription	*MED25*	↓	Colon	Mediator complex, subunit

0003197	Cofactors				Med25, PTOV activation and synapsin 2
ENSSSCG0000 0003200	Transcription Cofactors	*NUP62*	↓	Colon	Nucleoporin, NSP1-like, C-terminal
ENSSSCG0000 0003974	Transcription Cofactors	-	↑	Colon	CITED
ENSSSCG0000 0006014	Transcription Cofactors	*RAD21*	↓	Colon	Rad21/Rec8-like protein, C-terminal, eukaryotic
ENSSSCG0000 0006261	Transcription Cofactors	*TCEA1*	↓	Colon	Transcription factor IIS, N-terminal
ENSSSCG0000 0006493	Transcription Cofactors	*PMF1*	↓	Colon	Polyamine-modulated factor 1/Kinetochore protein NNF1
ENSSSCG0000 0006790	Transcription Cofactors	*WDR77*	↓	Colon	WD40 repeat
ENSSSCG0000 0007252	Transcription Cofactors	*DNMT3B*	↓	Colon	Zinc finger, FYVE/PHD-type
ENSSSCG0000 0007331	Transcription Cofactors	*RBL1*	↓	Colon	Retinoblastoma-associated protein, A-box
ENSSSCG0000 0008226	Transcription Cofactors	*POLR1A*	↓	Colon	RNA polymerase Rpb1, domain 5
ENSSSCG0000 0008624	Transcription Cofactors	-	↓	Liver	LNS2/PITP
ENSSSCG0000 0008788	Transcription Cofactors	-	↓	Colon	AAA+ ATPase domain
ENSSSCG0000 0009094	Transcription Cofactors	*CCNA2*	↓	Colon	Cyclin-like
ENSSSCG0000 0009569	Transcription Cofactors	*PSPC1*	↓	Colon	RNA recognition motif domain
ENSSSCG0000 0009746	Transcription Cofactors	*RAN*	↓	Colon	Small GTPase superfamily
ENSSSCG0000 0009873	Transcription Cofactors	*DTX1*	↓	Colon	Zinc finger, RING-type
ENSSSCG0000 0009876	Transcription Cofactors	*DDX54*	↓	Colon	Helicase superfamily 1/2, ATP-binding domain
ENSSSCG0000 0010493	Transcription Cofactors	-	↑	Colon	PDZ domain||ZASP||Zinc finger, LIM-type
ENSSSCG0000 0011548	Transcription Cofactors	*BRPF1*	↓	Colon	PWWP domain
ENSSSCG0000 0011746	Transcription Cofactors	*SKIL*	↑	Colon	Transforming protein Ski
ENSSSCG0000 0012056	Transcription Cofactors	*CHAF1B*	↓	Colon	G-protein, beta subunit
ENSSSCG0000	Transcription	*RBBP7*	↓	Colon	WD40 repeat

0012149	Cofactors				
ENSSSCG00000012241	Transcription Cofactors	*BCOR*	↓	Colon	Ankyrin repeat
ENSSSCG00000012793	Transcription Cofactors	*HCFC1*	↓	Colon	Fibronectin type III
ENSSSCG00000012904	Transcription Cofactors	*AIP*	↓	Colon	Tetratricopeptide repeat
ENSSSCG00000013019	Transcription Cofactors	*MEN1*	↓	Colon	Menin
ENSSSCG00000013746	Transcription Cofactors	*CALR*	↓	Jejunum	Calreticulin
ENSSSCG00000014670	Transcription Cofactors	*TRIM5*	↓	Jejunum	Concanavalin A-like lectin/glucanase domain
ENSSSCG00000015016	Transcription Cofactors	*POU2AF1*	↓	Jejunum	POU, class 2, associating factor 1
ENSSSCG00000015093	Transcription Cofactors	*CD3D*	↓	Jejunum	Phosphorylated immunoreceptor signalling ITAM
ENSSSCG00000015581	Transcription Cofactors	*CENPF*	↓	Jejunum	Centromere protein Cenp-F, leucine-rich repeat-containing domain
ENSSSCG00000016795	Transcription Cofactors	-	↓	Colon	Brain acid soluble protein 1
ENSSSCG00000017032	Transcription Cofactors	-	↓	Colon	Securin sister-chromatid separation inhibitor
ENSSSCG00000017428	Transcription Cofactors	*JUP*	↑	Colon	Armadillo-type fold
ENSSSCG00000017497	Transcription Cofactors	*ERBB2*	↑	Jejunum	Tyrosine protein kinase, EGF/ERB/XmrK receptor
ENSSSCG00000022101	Transcription Cofactors	*BRCA1*	↓	Colon	Breast cancer type 1 susceptibility protein (BRCA1)
ENSSSCG00000022508	Transcription Cofactors	*UIMC1*	↓	Colon	Ubiquitin interacting motif
ENSSSCG00000025116	Transcription Cofactors	*TONSL*	↓	Colon	Tetratricopeptide repeat-containing domain
ENSSSCG00000029275	Transcription Cofactors	*PPARGC1A*	↑	Colon	RNA recognition motif domain
ENSSSCG00000000697	Chromatin Remodeling Factors	*CHD4*	↓	Colon	Domain of unknown function DUF1086
ENSSSCG00000001074	Chromatin Remodeling Factors	*KDM1B*	↓	Colon	Pyridine nucleotide-disulphide oxidoreductase,

					FAD/NAD(P)-binding domain
ENSSSCG00000001958	Chromatin Remodeling Factors	*BAZ1A*	↓	Colon	DDT domain, subgroup
ENSSSCG00000003152	Chromatin Remodeling Factors	*RUVBL2*	↓	Colon	DNA helicase, DnaB-like, C-terminal
ENSSSCG00000004517	Chromatin Remodeling Factors	*CXXC1*	↓	Colon	Zinc finger, PHD-type
ENSSSCG00000006898	Chromatin Remodeling Factors	*MTF2*	↓	Colon	Zinc finger, FYVE/PHD-type
ENSSSCG00000009193	Chromatin Remodeling Factors	*SMARCAD1*	↓	Colon	SNF2-related, N-terminal domain
ENSSSCG00000009610	Chromatin Remodeling Factors	*NPM2*	↓	Colon	Nucleoplasmin core domain
ENSSSCG00000010070	Chromatin Remodeling Factors	*SMARCB1*	↓	Colon	SWI/SNF chromatin-remodeling complex, component hSNF5/Ini1
ENSSSCG00000011776	Chromatin Remodeling Factors	*YEATS2*	↓	Colon	YEATS
ENSSSCG00000013659	Chromatin Remodeling Factors	*DNMT1*	↓	Colon	S-adenosyl-L-methionine-dependent methyltransferase
ENSSSCG00000014915	Chromatin Remodeling Factors	-	↓	Colon	WD40-repeat-containing domain
ENSSSCG00000015288	Chromatin Remodeling Factors	*RBBP5*	↓	Colon	WD40 repeat
ENSSSCG00000016711	Chromatin Remodeling Factors	*CBX3*	↓	Colon	Chromo/chromo shadow domain
ENSSSCG00000017286	Chromatin Remodeling Factors	*SMARCD2*	↓	Colon	SWIB/MDM2 domain
ENSSSCG00000017744	Chromatin Remodeling	*SUZ12*	↓	Colon	Polycomb protein, VEFS-Box

	Factors				
ENSSSCG00000021363	Chromatin Remodeling Factors	*CHD3*	↓	Colon	CHD, N-terminal
ENSSSCG00000024261	Chromatin Remodeling Factors	*CBX2*	↓	Colon	Chromo domain-like
ENSSSCG00000025545	Chromatin Remodeling Factors	*HDAC6*	↑	Jejunum	Histone deacetylase domain
ENSSSCG00000028423	Chromatin Remodeling Factors	*RBBP4*	↓	Colon	WD40 repeat
ENSSSCG00000028840	Chromatin Remodeling Factors	*EZH2*	↓	Colon	WD repeat binding protein EZH2
ENSSSCG00000029450	Chromatin Remodeling Factors	-	↓	Colon	Histone deacetylase superfamily
