# Supplementary material for: Gene expression profiles of germ-free and conventional piglets from the same litter
Source: Sci Rep. 2018 Jul 16;8:10745. doi: 10.1038/s41598-018-29093-3 (PMC6048018; doi:10.1038/s41598-018-29093-3)
Supplement: Supplementary file 2 — Table S2 [file 41598_2018_29093_MOESM2_ESM.pdf]

Gene expression profiles of germ-free and conventional piglets from the same litter

Jing Sun[1,2,3,*], Hang Zhong[1,*], Lei Du[1], XiaoLei Li[1,4], Yuchun Ding[1,2,3], Haoran Cao[1,2,3], Zuohua Liu[1,2,3,+], and Liangpeng Ge[1,2,3,+]

Table S2. Top 30 DEGs in tissues by DEG identification.

Gene ID	Gene	Descriptions	logFC	FDR	Expression change in GF piglets[1]
DEGs cutoff: |logFC| > 1, FDR < 0.005					
Colon					
ENSSSCG00000013973	*LYPD8*	-	2.7414	0	UP
ENSSSCG00000005832	*MAMDC4*	Concanavalin A-like lectin/glucanase domain||Low-density lipoprotein (LDL) receptor class A repeat||MAM domain	3.5299	1.43E-273	UP
ENSSSCG00000001849	*ANPEP*	Peptidase M1, membrane alanine aminopeptidase, N-terminal	1.0984	1.06E-247	UP
ENSSSCG00000002981	*DMBT1*	SRCR-like domain||CUB domain||Zona pellucida domain||SRCR domain	5.1175	1.70E-238	UP
ENSSSCG00000009444	*OLFM4*	Olfactomedin-like domain	1.9876	1.08E-225	UP
ENSSSCG00000006933	*CLCA1*	Fibronectin type III||Calcium-activated chloride channel protein||Chloride channel calcium-activated||von Willebrand factor, type A	1.7345	3.26E-185	UP
ENSSSCG00000006142	*CA1*	Alpha carbonic anhydrase	12.137	4.76E-169	UP
ENSSSCG00000026605	*BPI*	Lipid-binding serum glycoprotein, N-terminal||Bactericidal permeability-increasing protein, alpha/beta domain||Lipid-binding serum glycoprotein, C-terminal	3.0125	1.24E-162	UP
ENSSSCG00000009327	*HMGB1*	High mobility group box domain	-1.5841	7.78E-160	DOWN
ENSSSCG00000003908	*TSPAN1*	Tetraspanin, EC2 domain||Tetraspanin||Tetraspanin/Peripherin	2.8497	2.07E-155	UP
ENSSSCG00000001379	*TUBB*	Beta tubulin||Tubulin/FtsZ, C-terminal||Tubulin/FtsZ, 2-layer sandwich domain||Gamma tubulin||Tubulin/FtsZ, GTPase domain||Tubulin||Epsilon	-1.6591	4.46E-151	DOWN

		tubulin||Misato Segment II tubulin-like domain||Alpha tubulin||Delta tubulin			
ENSSSCG00000015068	*APOA4*	Apolipoprotein A/E	2.3167	1.66E-138	UP
ENSSSCG00000026302	*MKI67*	Forkhead-associated (FHA) domain||SMAD/FHA domain||K167R	-2.2564	1.47E-137	DOWN
ENSSSCG00000002279	*GPX2*	Glutathione peroxidase||Thioredoxin-like fold	3.833	4.56E-132	UP
ENSSSCG00000012077	*MX1*	Dynamin GTPase effector||P-loop containing nucleoside triphosphate hydrolase||Dynamin central domain||Dynamin, GTPase domain||Dynamin superfamily	-3.4275	5.18E-125	DOWN[1]
ENSSSCG00000000190	*TUBA1B*	Tubulin||Alpha tubulin||Epsilon tubulin||Delta tubulin||Beta tubulin||Tubulin/FtsZ, 2-layer sandwich domain||Tubulin/FtsZ, C-terminal||Tubulin/FtsZ, GTPase domain	-1.9508	1.12E-124	DOWN
ENSSSCG00000021208	*SEPP1*	Selenoprotein P, N-terminal	1.0016	2.04E-119	UP
ENSSSCG00000030300	-		2.575	4.22E-115	UP
ENSSSCG00000005394	-		1.3865	4.02E-109	UP
ENSSSCG00000005055	*LGALS3*	Galectin, carbohydrate recognition domain||Concanavalin A-like lectin/glucanase domain	1.9938	1.11E-107	UP
ENSSSCG00000023333	-		1.7604	4.03E-107	UP
ENSSSCG00000022729	*KLHL6*	BTB/Kelch-associated||Kelch repeat type 1||Kelch repeat type 2	-3.1123	4.25E-104	DOWN
ENSSSCG00000017473	*TOP2A*	DNA topoisomerase, type IIA, subunit B, domain 2||Histidine kinase-like ATPase, C-terminal domain||DNA topoisomerase II, eukaryotic-type||Ribosomal protein S5 domain 2-type fold||DTHCT||DNA topoisomerase, type IIA||DNA topoisomerase, type IIA, subunit A/C-terminal||DNA topoisomerase, type IIA-like	-2.0429	7.61E-99	DOWN

		domain			
ENSSSCG00000023186	*CA4*	Alpha carbonic anhydrase	4.0086	2.21E-98	UP
ENSSSCG00000009412	*LCP1*	EF-hand domain‖Calponin homology domain	-2.0777	4.85E-97	DOWN
ENSSSCG00000010044	*IGLC*	CD80-like, immunoglobulin C2-set‖Immunoglobulin-like domain‖Immunoglobulin C1-set	-1.6665	7.70E-97	DOWN
Novel03259	*-//-*		-1.7696	2.25E-96	DOWN
ENSSSCG00000024634	*C10orf99*	-	3.0615	2.20E-95	UP
Jejunum					
ENSSSCG00000000492	*LYZ*	Glycoside hydrolase, family 22‖Lysozyme-like domain‖Transglycosylase SLT domain 1‖Glycoside hydrolase, family 22, lysozyme	-1.9853	0.00E+00	DOWN
ENSSSCG00000002981	*DMBT1*	SRCR-like domain‖CUB domain‖Zona pellucida domain‖SRCR domain	-2.3846	0	DOWN
ENSSSCG00000005394	-	Fructose-bisphosphate aldolase, class-I	1.6313	0	UP
ENSSSCG00000015068	*APOA4*	Apolipoprotein A/E	2.4748	0	UP
ENSSSCG00000015069	*APOC3*	Apolipoprotein CIII	3.9985	0	UP
ENSSSCG00000021208	*SEPP1*	Selenoprotein P, N-terminal	1.8362	0	UP
ENSSSCG00000021334	*CH242-307A4.1*	C-type lectin‖C-type lectin fold	-8.0451	0	DOWN
ENSSSCG00000023443	*REG3G*	C-type lectin‖C-type lectin fold	-9.0109	0	DOWN
ENSSSCG00000013551	-	Alpha-2-macroglobulin‖Alpha-2-macroglobulin, N-terminal‖Alpha-2-macroglobulin, thiol-ester bond-forming‖Terpenoid cyclases/protein prenyltransferase alpha-alpha toroid‖Alpha-macroglobulin complement component	-1.1506	7.85E-304	DOWN
ENSSSCG00000025543	*FABP2*	Cytosolic fatty-acid binding‖Lipocalin/cytosolic	1.525	1.81E-298	UP

		fatty-acid binding domain||Calycin-like			
ENSSSCG00000 009444	*OLFM4*	Olfactomedin-like domain	-1.2797	6.36E-281	DOWN
ENSSSCG00000 001231	*SLA-1*	MHC class I alpha chain, alpha1 alpha2 domains||MHC class I alpha chain||Immunoglobulin-like domain||MHC class I, alpha chain, C-terminal||MHC classes I/II-like antigen recognition protein||Immunoglobulin C1-set	-2.5314	1.90E-253	DOWN
ENSSSCG00000 018094	*CYTB*	Cytochrome b/b6, C-terminal||Di-haem cytochrome, transmembrane||Cytochrome b/b6, N-terminal	1.159	4.58E-220	UP
ENSSSCG00000 004682	*B2M*	Immunoglobulin C1-set||Immunoglobulin-like domain	-2.4528	6.87E-217	DOWN
ENSSSCG00000 001227	*-//-*		-3.5078	2.58E-206	DOWN
ENSSSCG00000 030385	-	Tissue inhibitor of metalloproteinases-like, OB-fold||Netrin domain||Netrin module, non-TIMP type||Alpha-macroglobulin, receptor-binding	-1.2561	1.93E-199	DOWN
ENSSSCG00000 030731	*TMP-SLA-2*	MHC class I, alpha chain, C-terminal	-2.4282	2.85E-191	DOWN
ENSSSCG00000 021006	-	Immunoglobulin C1-set||Immunoglobulin-like domain||CD80-like, immunoglobulin C2-set	-3.5465	6.80E-185	DOWN
ENSSSCG00000 015662	*C4BPA*	Sushi/SCR/CCP domain	-1.777	6.39E-176	DOWN
ENSSSCG00000 007507	*PCK1*	Phosphoenolpyruvate carboxykinase, GTP-utilising||Phosphoenolpyruvate carboxykinase, N-terminal	3.0456	1.26E-174	UP
ENSSSCG00000 010734	*OAT*	Aminotransferase class-III||Ornithine aminotransferase||Pyridoxal phosphate-dependent transferase	1.0491	5.77E-172	UP
ENSSSCG00000 010044	*IGLC*	CD80-like, immunoglobulin C2-set||Immunoglobulin-like	-2.3906	1.11E-160	DOWN

		domain‖Immunoglobulin C1-set			
ENSSSCG00000 001252	*UBD*	Ubiquitin‖Ubiquitin domain‖Ubiquitin-related domain	-4.0435	1.94E-155	DOWN
ENSSSCG00000 010893	*CFH*	Sushi/SCR/CCP domain	-2.1423	8.37E-150	DOWN
ENSSSCG00000 006932	-	Fibronectin type III‖Calcium-activated chloride channel protein‖von Willebrand factor, type A‖Chloride channel calcium-activated	1.5258	1.07E-147	UP
ENSSSCG00000 006924	*GBP1*	RHD3/Sey1‖Guanylate-binding protein, N-terminal‖Guanylate-binding protein, C-terminal‖P-loop containing nucleoside triphosphate hydrolase	-2.8736	2.35E-144	DOWN
ENSSSCG00000 002471	*ISG12(A)*	Interferon alpha-inducible protein 6/27	-2.4435	7.04E-143	DOWN
ENSSSCG00000 005395	-	Fructose-bisphosphate aldolase, class-I	1.7389	1.83E-140	UP
ENSSSCG00000 017163	*ENPP7*	Type I phosphodiesterase/nucleotide pyrophosphatase/phosphate transferase‖Alkaline-phosphatase-like, core domain	3.4487	3.38E-140	UP
ENSSSCG00000 012077	*MX1*	Dynamin GTPase effector‖P-loop containing nucleoside triphosphate hydrolase‖Dynamin central domain‖Dynamin, GTPase domain‖Dynamin superfamily	-3.5767	2.44E-125	DOWN
Spleen					
ENSSSCG00000 007978	*HBQ1*	Globin-like‖Haemoglobin, alpha‖Haemoglobin, pi‖Globin	1.4224	0	UP
ENSSSCG00000 008203	*IGKC*	Immunoglobulin C1-set‖CD80-like, immunoglobulin C2-set‖Immunoglobulin-like domain	-2.765	0	DOWN
ENSSSCG00000 008939	*JCHAIN*	-	-2.9567	0	DOWN
ENSSSCG00000 010044	*IGLC*	CD80-like, immunoglobulin C2-set‖Immunoglobulin-like domain‖Immunoglobulin C1-set	-3.1527	0	DOWN
ENSSSCG00000	*HBB*	Globin‖Myoglobin‖Haemoglobin,	1.2866	0	UP

014725		beta||Globin-like			
Novel03259	-//-		-2.7222	0	DOWN
ENSSSCG00000 003524	*C1QA*	Tumour necrosis factor-like domain||C1q domain||Collagen triple helix repeat	1.0198	2.75E-294	UP
ENSSSCG00000 004682	*B2M*	Immunoglobulin C1-set||Immunoglobulin-like domain	-1.9437	1.23E-240	DOWN
ENSSSCG00000 031054	*IGLV-8*	Immunoglobulin||Immunoglobulin V-set domain||Immunoglobulin subtype||Immunoglobulin-like domain	-3.8982	1.69E-136	DOWN
ENSSSCG00000 024853	-//-		-1.3537	1.60E-117	DOWN
ENSSSCG00000 030927	*IGKV-7*	Immunoglobulin-like domain||Immunoglobulin subtype||Immunoglobulin V-set domain	-4.1276	1.66E-117	DOWN
ENSSSCG00000 001227	-//-		-2.2976	4.16E-115	DOWN
ENSSSCG00000 001231	*SLA-1*	MHC class I alpha chain, alpha1 alpha2 domains||MHC class I alpha chain||Immunoglobulin-like domain||MHC class I, alpha chain, C-terminal||MHC classes I/II-like antigen recognition protein||Immunoglobulin C1-set	-1.3826	2.12E-113	DOWN
ENSSSCG00000 015716	*MARCO*	SRCR domain||Collagen triple helix repeat||SRCR-like domain	1.4885	2.35E-94	UP
ENSSSCG00000 003526	*C1QB*	Collagen triple helix repeat||Tumour necrosis factor-like domain||C1q domain	1.7655	6.71E-75	UP
ENSSSCG00000 030825	*IGLV-9*	Immunoglobulin subtype||Immunoglobulin||Immunoglobulin V-set domain||Immunoglobulin-like domain	-5.3144	3.30E-72	DOWN
ENSSSCG00000 031037	*IGLV-8*	Immunoglobulin||Immunoglobulin V-set domain||Immunoglobulin subtype||Immunoglobulin-like domain	-2.8324	3.28E-62	DOWN
ENSSSCG00000 004687	-	Immunoglobulin-like domain||Immunoglobulin C1-set	-1.3058	1.33E-58	DOWN
ENSSSCG00000	*MX1*	Dynamin GTPase effector||P-loop	-1.7668	2.09E-	DOWN

012077		containing nucleoside triphosphate hydrolase||Dynamin central domain||Dynamin, GTPase domain||Dynamin superfamily		54	
ENSSSCG00000 006455	*CD5L*	SRCR-like domain||SRCR domain	-1.4307	9.84E-53	DOWN
ENSSSCG00000 030868	*IGLV-10*	Immunoglobulin V-set domain||Immunoglobulin||Immunoglobulin subtype||Immunoglobulin-like domain	-4.1112	3.85E-51	DOWN
ENSSSCG00000 017358	*SLC4A1*	Phosphotransferase/anion transporter||Bicarbonate transporter, C-terminal||Bicarbonate transporter, eukaryotic||Anion exchange protein||Band 3 cytoplasmic domain	4.8505	1.02E-44	UP
ENSSSCG00000 027982	*ISG15*	Ubiquitin domain||Ubiquitin||Rad60/SUMO-like domain||Ubiquitin-related domain	-2.5716	3.70E-40	DOWN
Novel00917	-//-		-5.5231	1.43E-35	DOWN
ENSSSCG00000 002471	*ISG12(A)*	Interferon alpha-inducible protein 6/27	-1.8671	3.44E-33	DOWN
ENSSSCG00000 006979	*MSR1*	SRCR-like domain||Collagen triple helix repeat||STAT transcription factor, coiled coil||Macrophage scavenger receptor||SRCR domain	2.7676	4.98E-30	UP
ENSSSCG00000 021356	-//-		-2.0081	4.40E-29	DOWN
ENSSSCG00000 009240	*PLAC8*	PLAC8 motif-containing protein	-1.6841	3.53E-26	DOWN
ENSSSCG00000 029605	-//-		2.0056	4.12E-25	UP
ENSSSCG00000 029239	*MZB1*		-2.0854	3.88E-21	DOWN
Liver					
ENSSSCG00000 003088	*APOE*	Apolipoprotein A/E	-1.0626	0	DOWN
ENSSSCG00000 008997	*FGB*	Fibrinogen, alpha/beta/gamma chain, C-terminal globular domain||Fibrinogen,	1.177	0	UP

		alpha/beta/gamma chain, coiled coil domain			
ENSSSCG00000 015068	*APOA4*	Apolipoprotein A/E	-1.0684	0	DOWN
ENSSSCG00000 024314	-	Fibrinogen, alpha/beta/gamma chain, C-terminal globular domain||Fibrinogen, alpha/beta/gamma chain, coiled coil domain	1.8529	0	UP
ENSSSCG00000 030371	-	Serpin domain	1.5744	0	UP
Novel02761	-//-		1.3336	0	UP
ENSSSCG00000 011700	*CP*	Cupredoxin||Multicopper oxidase, type 2||Multicopper oxidase, type 1||Multicopper oxidase, type 3	1.2037	3.94E-281	UP
ENSSSCG00000 023305	-	Metallothionein||Metallothionein, vertebrate||Metallothionein domain	3.4875	1.60E-265	UP
ENSSSCG00000 010488	-	Cytochrome P450, E-class, group IV||Cytochrome P450, E-class, group I||Cytochrome P450, B-class||Cytochrome P450	-3.1585	2.23E-264	DOWN
ENSSSCG00000 002486	-	Serpin domain	1.5219	2.73E-244	UP
ENSSSCG00000 008595	-	Vitellinogen, superhelical||Lipid transport protein, N-terminal||Lipid transport protein, beta-sheet shell||Vitellinogen, open beta-sheet||Lipid transport, open beta-sheet	2.5855	6.88E-216	UP
ENSSSCG00000 011801	*HRG*	Cystatin domain	-1.2291	2.86E-183	DOWN
ENSSSCG00000 016402	*AGXT*	Pyridoxal phosphate-dependent transferase||Aminotransferase class V domain	-2.3027	1.52E-181	DOWN
ENSSSCG00000 005701	*ASS1*	Argininosuccinate synthase||Asparagine synthase||Queuosine biosynthesis protein QueC	-1.4285	1.25E-170	DOWN
ENSSSCG00000 001901	*CYP1A2*	Cytochrome P450||Cytochrome P450, E-class, group I, CYP1||Cytochrome P450, E-class, group IV||Cytochrome P450, E-class, group I	-3.2813	2.78E-158	DOWN
ENSSSCG00000	*CRP*	Pentaxin-related||Concanavalin	1.4635	1.56E-	UP

006403		A-like lectin/glucanase domain		139	
ENSSSCG00000 011453	*ITIH4*	VIT domain||von Willebrand factor, type A	1.111	5.76E-139	UP
ENSSSCG00000 024853	*-//-*		-1.8364	5.14E-131	DOWN
ENSSSCG00000 028901	-	Fibrinogen, alpha/beta/gamma chain, C-terminal globular domain	1.5151	1.81E-121	UP
ENSSSCG00000 002886	*HAMP*	Hepcidin	2.2391	2.29E-109	UP
ENSSSCG00000 028758	*LBP*	Lipid-binding serum glycoprotein, C-terminal||Bactericidal permeability-increasing protein, alpha/beta domain||Lipid-binding serum glycoprotein, N-terminal	1.588	9.63E-89	UP
ENSSSCG00000 023684	-	Metallothionein domain||Metallothionein, vertebrate||Metallothionein	1.079	1.09E-71	UP
ENSSSCG00000 010368	*GLUD1*	Glutamate/phenylalanine/leucine/valine dehydrogenase, dimerisation domain||Glutamate/phenylalanine/leucine/valine dehydrogenase, C-terminal||Glutamate/phenylalanine/leucine/valine dehydrogenase	1.0086	8.26E-62	UP
ENSSSCG00000 001231	*SLA-1*	MHC class I alpha chain, alpha1 alpha2 domains||MHC class I alpha chain||Immunoglobulin-like domain||MHC class I, alpha chain, C-terminal||MHC classes I/II-like antigen recognition protein||Immunoglobulin C1-set	-1.8173	1.59E-61	DOWN
ENSSSCG00000 018069	*ND2*	NADH:ubiquinone oxidoreductase, chain 2||NADH dehydrogenase subunit 2, C-terminal||NADH:quinone oxidoreductase/Mrp antiporter, membrane subunit	1.0619	5.45E-60	UP
ENSSSCG00000 024929	-	Fibrinogen, alpha/beta/gamma chain, coiled coil domain	1.261	3.95E-59	UP
ENSSSCG00000 027982	*ISG15*	Ubiquitin domain||Ubiquitin||Rad60/SUMO-like domain||Ubiquitin-related domain	-2.9022	1.74E-53	DOWN
ENSSSCG00000 002471	*ISG12(A)*	Interferon alpha-inducible protein 6/27	-2.2218	3.27E-52	DOWN

ENSSSCG00000 012676	*MBNL3*	Zinc finger, CCCH-type||-	1.6767	7.15E-50	UP
ENSSSCG00000 004682	*B2M*	Immunoglobulin C1-set||Immunoglobulin-like domain	-1.4272	4.87E-48	DOWN
Oral mucosa					
ENSSSCG00000 000245	-	Keratin, type II||Intermediate filament protein||Prefoldin	1.0436	0	UP
ENSSSCG00000 028522	*KRT10*	Intermediate filament protein||Prefoldin||Keratin, type I	-1.4423	0	DOWN
ENSSSCG00000 000251	*KRT1*	Prefoldin||Intermediate filament protein||Keratin, type II	-2.5677	1.42E-278	DOWN
ENSSSCG00000 006589	*S100A12*	EF-hand domain||S100/CaBP-9k-type, calcium binding, subdomain	1.2891	2.80E-153	UP
Novel01936	*S100A12*	EF-hand domain||S100/CaBP-9k-type, calcium binding, subdomain	1.5102	1.06E-109	UP
ENSSSCG00000 024610	*KRT4*	Intermediate filament protein||Keratin, type II	2.2736	2.69E-103	UP
Novel00535	*-//-*		1.8965	1.02E-76	UP
ENSSSCG00000 004682	*B2M*	Immunoglobulin C1-set||Immunoglobulin-like domain	-2.1063	1.47E-74	DOWN
ENSSSCG00000 012077	*MX1*	Dynamin GTPase effector||P-loop containing nucleoside triphosphate hydrolase||Dynamin central domain||Dynamin, GTPase domain||Dynamin superfamily	-3.6491	3.83E-73	DOWN
ENSSSCG00000 006590	*S100A8*	S100/CaBP-9k-type, calcium binding, subdomain||EF-hand domain	1.3148	2.22E-69	UP
Novel02034	*-//-*		1.064	2.18E-66	UP
ENSSSCG00000 002471	*ISG12(A)*	Interferon alpha-inducible protein 6/27	-3.0707	4.71E-58	DOWN
Novel02149	*-//-*		-1.5484	9.90E-56	DOWN
ENSSSCG00000 027982	*ISG15*	Ubiquitin domain||Ubiquitin||Rad60/SUMO-like domain||Ubiquitin-related domain	-4.0598	4.56E-47	DOWN
Novel02033	*-//-*		-1.5038	4.58E-	DOWN

				46	
ENSSSCG00000 006588	*S100A9*	EF-hand domain||S100/CaBP-9k-type, calcium binding, subdomain	1.2124	2.02E-45	UP
ENSSSCG00000 017146	*RNF213*	Zinc finger, RING-type||P-loop containing nucleoside triphosphate hydrolase||AAA+ ATPase domain||-	-2.0515	9.99E-40	DOWN
ENSSSCG00000 006592	*S100A7*	S100/CaBP-9k-type, calcium binding, subdomain||EF-hand domain	1.1492	1.19E-33	UP
ENSSSCG00000 001453	*SLA-DRA*	Immunoglobulin-like domain||MHC classes I/II-like antigen recognition protein||MHC class II, alpha chain, N-terminal||Immunoglobulin C1-set	-1.2498	2.04E-28	DOWN
ENSSSCG00000 030408	*DDX58*	Helicase, C-terminal||Helicase/UvrB, N-terminal||DEAD/DEAH box helicase domain||Death-like domain||C-terminal domain of RIG-I||P-loop containing nucleoside triphosphate hydrolase||Helicase superfamily 1/2, ATP-binding domain	-3.1698	3.79E-25	DOWN
ENSSSCG00000 023406	-//-		2.6244	6.05E-25	UP
ENSSSCG00000 000492	*LYZ*	Glycoside hydrolase, family 22||Lysozyme-like domain||Transglycosylase SLT domain 1||Glycoside hydrolase, family 22, lysozyme	1.5043	6.31E-25	UP
ENSSSCG00000 021591	-	RNA recognition motif domain	1.124	3.63E-20	UP
ENSSSCG00000 009881	*OAS2*	Polymerase, nucleotidyl transferase domain||2-5-oligoadenylate synthetase, N-terminal||2'-5'-oligoadenylate synthetase 1, domain 2/C-terminal	-2.317	7.57E-20	DOWN
ENSSSCG00000 001227	-//-		-2.0611	1.48E-19	DOWN
ENSSSCG00000 023379	*UBE2L6*	Ubiquitin-conjugating enzyme E2||Ubiquitin-conjugating	-2.4008	7.95E-19	DOWN

		enzyme/RWD-like			
ENSSSCG00000030031	*FLG2*	Filaggrin‖S100/CaBP-9k-type, calcium binding, subdomain‖EF-hand domain	-2.5723	1.20E-17	DOWN
ENSSSCG00000001231	*SLA-1*	MHC class I alpha chain, alpha1 alpha2 domains‖MHC class I alpha chain‖Immunoglobulin-like domain‖MHC class I, alpha chain, C-terminal‖MHC classes I/II-like antigen recognition protein‖Immunoglobulin C1-set	-1.1378	2.34E-17	DOWN
ENSSSCG00000010453	*IFIT1*	Tetratricopeptide repeat‖Tetratricopeptide repeat 2‖Tetratricopeptide repeat 1‖Tetratricopeptide repeat-containing domain	-2.9008	8.77E-17	DOWN
ENSSSCG00000000774	*IFI44L*	P-loop containing nucleoside triphosphate hydrolase‖TLDc domain	-3.0542	1.07E-16	DOWN

[1]Differential expressed genes in the above top 30 DEGs list were marked in yellow when they also belonged to the 14 comment elements in Figure 1C.
