# Supplementary material for: Gene expression profiles of germ-free and conventional piglets from the same litter
Source: Sci Rep. 2018 Jul 16;8:10745. doi: 10.1038/s41598-018-29093-3 (PMC6048018; doi:10.1038/s41598-018-29093-3)
Supplement: Supplementary file 3 — Table S3 [file 41598_2018_29093_MOESM3_ESM.pdf]

Gene expression profiles of germ-free and conventional piglets from the same litter

Jing Sun[1,2,3,*], Hang Zhong[1,*], Lei Du[1], XiaoLei Li[1,4], Yuchun Ding[1,2,3], Haoran Cao[1,2,3], Zuohua Liu[1,2,3,+], and Liangpeng Ge[1,2,3,+]

Table S3. The most Enriched pathways (Gene ontology, biological process) of DEGs in each tissue.

Term		Count	Corrected P-value	%[1]	Up%	Down%
Jejunum (JEJ)						
GO:0006955	immune response	79	2.91E-23	16.02%	1.42%	14.60%
GO:0002376	immune system process	107	4.12E-21	21.70%	2.03%	19.68%
GO:0045087	innate immune response	39	1.48E-13	7.91%	0.41%	7.51%
GO:0006952	defense response	63	1.12E-12	12.78%	0.81%	11.97%
GO:0044281	small molecule metabolic process	82	2.42E-11	16.63%	11.56%	5.07%
GO:0002684	positive regulation of immune system process	43	4.24E-10	8.72%	0.61%	8.11%
GO:0002252	immune effector process	40	4.96E-10	8.11%	0.41%	7.71%
GO:0098542	defense response to other organism	33	6.35E-10	6.69%	0.20%	6.49%
GO:0002682	regulation of immune system process	54	1.54E-09	10.95%	1.22%	9.74%
GO:0009615	response to virus	28	2.05E-09	5.68%	0.00%	5.68%
GO:0009607	response to biotic stimulus	43	8.39E-09	8.72%	0.20%	8.52%
GO:0051707	response to other organism	41	1.47E-08	8.32%	0.20%	8.11%
GO:0043207	response to external biotic stimulus	41	1.47E-08	8.32%	0.20%	8.11%
GO:0007159	leukocyte cell-cell adhesion	31	9.18E-08	6.29%	0.61%	5.68%
GO:0051607	defense response to virus	23	1.05E-07	4.67%	0.00%	4.67%
GO:0019752	carboxylic acid metabolic process	46	1.24E-07	9.33%	7.51%	1.83%
GO:0044710	single-organism metabolic process	157	1.27E-07	31.85%	17.04%	14.81%
GO:0006082	organic acid metabolic process	49	1.27E-07	9.94%	8.11%	1.83%
GO:0006950	response to stress	105	1.67E-07	21.30%	3.65%	17.65%
GO:0050778	positive regulation of immune response	29	1.69E-07	5.88%	0.20%	5.68%
GO:0043436	oxoacid metabolic process	48	1.73E-07	9.74%	7.91%	1.83%
Colon						
GO:0002376	immune system process	233	2.91E-22	14.68%	4.73%	9.96%
GO:0022402	cell cycle process	158	2.20E-21	9.96%	1.95%	8.00%
GO:0007049	cell cycle	186	2.64E-21	11.72%	2.71%	9.01%
GO:0006950	response to stress	318	1.34E-18	20.04%	7.75%	12.29%
GO:0000278	mitotic cell cycle	118	1.42E-18	7.44%	1.45%	5.99%
GO:1903047	mitotic cell cycle process	102	2.11E-18	6.43%	0.88%	5.55%

GO:0006955	immune response	138	4.94E-18	8.70%	2.27%	6.43%
GO:0048519	negative regulation of biological process	397	9.45E-18	25.02%	10.84%	14.18%
GO:0048518	positive regulation of biological process	463	1.31E-16	29.17%	13.36%	15.82%
GO:0048523	negative regulation of cellular process	367	7.24E-16	23.13%	10.14%	12.98%
GO:0051276	chromosome organization	138	1.63E-14	8.70%	0.57%	8.13%
GO:0016043	cellular component organization	499	7.63E-14	31.44%	13.11%	18.34%
GO:0008283	cell proliferation	179	7.84E-14	11.28%	4.66%	6.62%
GO:0002682	regulation of immune system process	128	8.08E-14	8.07%	2.71%	5.36%
GO:0048522	positive regulation of cellular process	395	1.14E-13	24.89%	11.78%	13.11%
GO:0007059	chromosome segregation	59	8.19E-13	3.72%	0.13%	3.59%
GO:0045321	leukocyte activation	92	1.05E-12	5.80%	1.39%	4.41%
GO:0002252	immune effector process	84	1.71E-12	5.29%	1.20%	4.10%
GO:0002684	positive regulation of immune system process	91	1.78E-12	5.73%	1.64%	4.10%
GO:0043933	macromolecular complex subunit organization	236	2.96E-12	14.87%	4.79%	10.08%
GO:0051726	regulation of cell cycle	115	3.44E-12	7.25%	1.89%	5.36%
GO:0071840	cellular component organization or biogenesis	504	4.16E-12	31.76%	13.17%	18.59%
GO:0010564	regulation of cell cycle process	77	4.69E-12	4.85%	0.88%	3.97%
GO:0042127	regulation of cell proliferation	146	7.90E-12	9.20%	3.97%	5.23%
Spleen						
GO:0002376	immune system process	38	2.51E-12	33.93%	10.71%	23.21%
GO:0006955	immune response	24	7.50E-08	21.43%	2.68%	18.75%
GO:0098542	defense response to other organism	16	1.06E-07	14.29%	2.68%	11.61%
GO:0051707	response to other organism	18	1.43E-06	16.07%	2.68%	13.39%
GO:0043207	response to external biotic stimulus	18	1.43E-06	16.07%	2.68%	13.39%
GO:0009607	response to biotic stimulus	18	3.34E-06	16.07%	2.68%	13.39%
GO:0006952	defense response	21	1.20E-05	18.75%	4.46%	14.29%
GO:0051607	defense response to virus	11	1.56E-05	9.82%	0.89%	8.93%
GO:0032020	ISG15-protein conjugation	4	2.00E-05	3.57%	0.00%	3.57%
GO:0045071	negative regulation of viral genome replication	6	2.12E-05	5.36%	0.89%	4.46%
GO:0002520	immune system development	17	8.16E-05	15.18%	8.04%	7.14%
GO:0009615	response to virus	11	8.83E-05	9.82%	0.89%	8.93%

GO:0002252	immune effector process	14	0.000134	12.50%	1.79%	10.71%
GO:0048534	hematopoietic or lymphoid organ development	16	0.00017	14.29%	7.14%	7.14%
GO:0044764	multi-organism cellular process	11	0.000211	9.82%	3.57%	6.25%
GO:0045087	innate immune response	12	0.000211	10.71%	0.89%	9.82%
GO:0045069	regulation of viral genome replication	6	0.000211	5.36%	0.89%	4.46%
GO:0015669	gas transport	4	0.000211	3.57%	3.57%	0.00%
GO:0043900	regulation of multi-organism process	11	0.000266	9.82%	1.79%	8.04%
GO:1903901	negative regulation of viral life cycle	6	0.000329	5.36%	0.89%	4.46%
GO:0048525	negative regulation of viral process	6	0.00035	5.36%	0.89%	4.46%
GO:0019079	viral genome replication	6	0.000914	5.36%	0.89%	4.46%
GO:0043901	negative regulation of multi-organism process	7	0.000941	6.25%	0.89%	5.36%
GO:0030097	hemopoiesis	14	0.001341	12.50%	5.36%	7.14%
GO:0002682	regulation of immune system process	16	0.001836	14.29%	1.79%	12.50%
GO:0051704	multi-organism process	21	0.003169	18.75%	5.36%	13.39%
Liver						
GO:0044281	small molecule metabolic process	48	7.65E-10	22.54%	7.98%	14.55%
GO:0055114	oxidation-reduction process	37	2.70E-09	17.37%	7.98%	9.39%
GO:0044710	single-organism metabolic process	88	3.03E-09	41.31%	19.25%	22.07%
GO:0019752	carboxylic acid metabolic process	30	3.38E-08	14.08%	4.23%	9.86%
GO:0043436	oxoacid metabolic process	30	2.14E-07	14.08%	4.23%	9.86%
GO:0006082	organic acid metabolic process	30	3.31E-07	14.08%	4.23%	9.86%
GO:0006629	lipid metabolic process	32	5.61E-07	15.02%	5.63%	9.39%
GO:0009056	catabolic process	43	6.59E-06	20.19%	8.92%	11.27%
GO:0008202	steroid metabolic process	13	6.59E-06	6.10%	2.35%	3.76%
GO:0044283	small molecule biosynthetic process	17	6.59E-06	7.98%	2.82%	5.16%
GO:0044248	cellular catabolic process	39	6.68E-06	18.31%	7.51%	10.80%
GO:0044711	single-organism biosynthetic process	32	4.46E-05	15.02%	5.63%	9.39%
GO:0044255	cellular lipid metabolic process	24	6.01E-05	11.27%	4.23%	7.04%
GO:0010876	lipid localization	15	6.01E-05	7.04%	4.23%	2.82%
GO:0006952	defense response	27	0.000135	12.68%	5.16%	7.51%

GO:1901575	organic substance catabolic process	35	0.000148	16.43%	7.98%	8.45%
GO:1901617	organic hydroxy compound biosynthetic process	10	0.000168	4.69%	1.41%	3.29%
GO:0009607	response to biotic stimulus	21	0.000205	9.86%	2.82%	7.04%
GO:0006066	alcohol metabolic process	12	0.000205	5.63%	1.41%	4.23%
Oral mucosa						
GO:0034340	response to type I interferon	8	9.42E-17	8.99%	0.00%	8.99%
GO:0071357	cellular response to type I interferon	7	9.42E-17	7.87%	0.00%	7.87%
GO:0009607	response to biotic stimulus	23	5.76E-13	25.84%	2.25%	23.60%
GO:0051607	defense response to virus	16	5.76E-13	17.98%	0.00%	17.98%
GO:0002376	immune system process	33	2.63E-12	37.08%	7.87%	29.21%
GO:0006952	defense response	26	2.63E-12	29.21%	6.74%	22.47%
GO:0098542	defense response to other organism	18	3.69E-12	20.22%	1.12%	19.10%
GO:0009615	response to virus	16	7.44E-12	17.98%	0.00%	17.98%
GO:0006955	immune response	25	7.44E-12	28.09%	4.49%	23.60%
GO:0002252	immune effector process	19	5.30E-11	21.35%	2.25%	19.10%
GO:0045087	innate immune response	17	6.41E-11	19.10%	2.25%	16.85%
GO:0051707	response to other organism	20	7.87E-11	22.47%	2.25%	20.22%
GO:0043207	response to external biotic stimulus	20	7.87E-11	22.47%	2.25%	20.22%
GO:0031347	regulation of defense response	15	4.01E-08	16.85%	4.49%	12.36%
GO:0060337	type I interferon signaling pathway	6	1.84E-07	6.74%	0.00%	6.74%
GO:0009605	response to external stimulus	26	1.92E-07	29.21%	6.74%	22.47%
GO:0043900	regulation of multi-organism process	12	2.70E-06	13.48%	1.12%	12.36%
GO:0045071	negative regulation of viral genome replication	6	2.95E-06	6.74%	0.00%	6.74%
GO:0032020	ISG15-protein conjugation	4	3.47E-06	4.49%	0.00%	4.49%
GO:0006950	response to stress	31	5.17E-06	34.83%	10.11%	24.72%
GO:0043901	negative regulation of multi-organism process	8	1.22E-05	8.99%	1.12%	7.87%
GO:0045069	regulation of viral genome replication	6	4.27E-05	6.74%	0.00%	6.74%
GO:0002831	regulation of response to biotic stimulus	8	5.81E-05	8.99%	1.12%	7.87%
GO:0051704	multi-organism process	21	7.68E-05	23.60%	3.37%	20.22%
GO:1903901	negative regulation of viral life cycle	6	7.68E-05	6.74%	0.00%	6.74%

GO:0048525	negative regulation of viral process	6	8.29E-05	6.74%	0.00%	6.74%
GO:0035455	response to interferon-alpha	4	0.000127	4.49%	0.00%	4.49%
GO:0050688	regulation of defense response to virus	7	0.000127	7.87%	0.00%	7.87%
GO:0019079	viral genome replication	6	0.000188	6.74%	0.00%	6.74%
GO:0034097	response to cytokine	12	0.000188	13.48%	2.25%	11.24%

[1]% (percentage) = the number of genes for each term / total number of differentially expressed genes (DEGs) in each tissue. The percentage that greater than 15% was marked in red.

Count: number of DEGs that belongs to the term.

Corrected *P*-value: enrichment p-value measured by Fisher exact test.
