# Supplementary material for: Gene expression profiles of germ-free and conventional piglets from the same litter
Source: Sci Rep. 2018 Jul 16;8:10745. doi: 10.1038/s41598-018-29093-3 (PMC6048018; doi:10.1038/s41598-018-29093-3)
Supplement: Supplementary file 4 — Table S4 [file 41598_2018_29093_MOESM4_ESM.pdf]

Gene expression profiles of germ-free and conventional piglets from the same litter

Jing Sun[1,2,3,*], Hang Zhong[1,*], Lei Du[1], Xiaolei Li[1,4], Yuchun Ding[1,2,3], Haoran Cao[1,2,3], Zuohua Liu[1,2,3,+], and Liangpeng Ge[1,2,3,+]

Table S4. Primers of real-time quantitative PCR (QPCR).

Gene ID	Gene name	Annealing (℃)	Product (bp)	Rev primer	For primer	Article ID
ENSSSCG00000007807	*CD19*	60	84	GTGACTGGAAGAGGACCTGC	CGGTGGAATGCTTCAGACCT	10.1016/j.vetimm.2016.04.003
ENSSSCG00000003608	*LCK*	55	176	CACCAGGCTCCCATTCTC	ACACGAAGGTGGCAGTAAA	10.1007/s11033-011-1183-6
ENSSSCG00000011877	*CD86*	50	256	GAAGAGACACCCTGATTGATAC	GTTCCTATCCACCAGATGAGT	10.13473/j.cnki.issn.1002-3186.2006.02.009
ENSSSCG00000016057	*STAT-1*	60	74	GAAAACGGATGGTGGCAAAC	TCTGGCACAGTGGCTAGAAAATC	10.1016/j.intimp.2016.04.032
ENSSSCG00000025652	*CDH1*	64	117	GTTGTCCCGGGTGTCATCTT	ATTCTGGGAGGCATCCTTGC	10.1371/journal.pone.0158046
ENSSSCG00000027982	*ISG15*	60	84	GTCCCAGGGCCACCACATAG	CTGGAGGGTGGGGAGGGTAG	10.1371/journal.pone.0138653
ENSSSCG00000004682	*B2M*	60	166	CCAGATACATAGCAGTTCAGG	TTCACACCGCTCCAGTAG	10.1016/j.jbiotec.2011.04.002
ENSSSCG00000008977	*CXCL10*	58	84	GGCACTACTGATAAGGATGGG	CAGTTAGGGCTTGATGTATGGT	Master’s degree dissertation1
ENSSSCG00000008217	*CD8A*	61	103	TATCGTAGTCGGCGTCTTGG	TCCGATATGATAAAGACGAGCA	Master’s degree dissertation2
ENSSSCG00000025652	*ITGB2*	60	150-200	CCTCCGTCTGGAACTGGTTG	CGCATCGGCTTTGGGTCTT	Doctor’s degree dissertation3
ENSSSCG00000002383	*FOS*	65	374	CACCCATCTTATTCCTTTCCCTT	CCTTTCCTACTACCATTCCCC	10.16656/j.issn.1673-4696.2014.09.017
Housekeeping gene	*β-actin*	60	71	CCACGTCGCACTTCATGATC	CTCCTTCCTGGGCATGGA	10.3969/j.issn.1008-0589.2011.11.11

References

1. Xia, Y. Expression of CXCL10/CXCR3 in PRRSV infected porcine lung tissues and the macrophages Master's degree thesis, Huazhong Agricultural University, (2016).

2. Chen, R. Study on CD8a gene expression in bone marrow of weaned pig infected by PRRSV Master's degree thesis, Shanxi Agricultural University, (2014).

3. Zeng, L. L-arginine affects arginine transportation, cell proliferation and gene expression in intestinal porcine epithelial cells (IPEC-1) Doctor degree thesis, Hunan Agricultural University, (2012).
